# Gasdermin-D activation promotes NLRP3 activation and host resistance to *Leishmania* infection

**DOI:** 10.1038/s41467-023-36626-6

**Published:** 2023-02-24

**Authors:** Keyla S. G. de Sá, Luana A. Amaral, Tamara S. Rodrigues, Adriene Y. Ishimoto, Warrison A. C. de Andrade, Leticia de Almeida, Felipe Freitas-Castro, Sabrina S. Batah, Sergio C. Oliveira, Mônica T. Pastorello, Alexandre T. Fabro, Dario S. Zamboni

**Affiliations:** 1 Departamento de Biologia Celular e Molecular e Bioagentes Patogênicos, Faculdade de Medicina de Ribeirão Preto, Universidade de São Paulo, Ribeirão Preto, SP 14049-900 Brazil; 2 Departamento de Patologia e Medicina Legal, Faculdade de Medicina de Ribeirão Preto, Universidade de São Paulo, Ribeirão Preto, SP 14049-900 Brazil; 3 Departamento de Bioquímica e Imunologia, Universidade Federal de Minas Gerais, Belo Horizonte, MG 31270-901 Brazil; 4 Serviço de Patologia do Hospital das Clínicas da Faculdade de Medicina de Ribeirão Preto, Universidade de São Paulo, Ribeirão Preto, SP 14049-900 Brazil

**Keywords:** Parasite host response, Parasitic infection, NOD-like receptors

## Abstract

Intracellular parasites from the *Leishmania* genus cause Leishmaniasis, a disease affecting millions of people worldwide. NLRP3 inflammasome is key for disease outcome, but the molecular mechanisms upstream of the inflammasome activation are still unclear. Here, we demonstrate that despite the absence of pyroptosis, Gasdermin-D (GSDMD) is active at the early stages of *Leishmania* infection in macrophages, allowing transient cell permeabilization, potassium efflux, and NLRP3 inflammasome activation. Further, GSDMD is processed into a non-canonical 25 kDa fragment. *Gsdmd*^*–/–*^ macrophages and mice exhibit less NLRP3 inflammasome activation and are highly susceptible to infection by several *Leishmania* species, confirming the role of GSDMD for inflammasome-mediated host resistance. Active NLRP3 inflammasome and GSDMD are present in skin biopsies of patients, demonstrating activation of this pathway in human leishmaniasis. Altogether, our findings reveal that *Leishmania* subverts the normal functions of GSDMD, an important molecule to promote inflammasome activation and immunity in Leishmaniasis.

## Abstract (web summary)

Here, de Sá et al. show that Gasdermin-D is transiently activated in Leishmania-infected macrophages and promotes NLRP3 inflammasome activation, but not cell death. Gasdermin-D is cleaved into a noncanonical fragment, indicating that Leishmania subverts Gasdermin-D-mediated host response to establish leishmaniasis.

## Introduction

Leishmaniasis is a neglected tropical disease that mainly affects less privileged populations in tropical and subtropical countries in Africa, Asia, and Latin America^1,2^. More than 20 species can cause Leishmaniasis in humans. This disease’s clinical manifestations may be visceral or cutaneous, ranging from single and painless lesions to diffuse or mucocutaneous forms^1,3,4^. It is estimated that about 2 million new cases appear yearly, and 12 million patients have active disease^1,2^.

Once in the mammalian hosts, *Leishmania* replicates in professional phagocytes, and it is well accepted that cell death of the host cells is an effective mechanism to impair *Leishmania* replication. Accordingly, inhibition of apoptosis in *Leishmania* infection has been reported in a broad range of species and in many cell types^5–10^. Thus, early after infection, the parasite manipulates the cell death machinery to prevent host cell death and secure the formation of the parasitophorous vacuoles that support parasite replication.

Regardless of cell death, activation of innate immune receptors is a critical mechanism for the restriction of *Leishmania* infection (reviewed in ref. ^11^). Members of the endosomal Toll-like receptors (TLR3, 7, and 9) and the inflammasomes are among the innate immune receptors activated during *Leishmania* infection^12–18^. The inflammasome is a protein complex composed of a sensor protein (such as NLRP3), an adapter protein (such as ASC protein), and an inflammatory caspase, such as caspase-1 and caspase-4 (human) and caspase-11 (mice) (reviewed in ref. ^19^).

Activation of inflammatory caspases through the inflammasome can lead to inflammatory cell death, known as pyroptosis, a process that is important for the elimination of intracellular pathogens (reviewed in ref. ^20^). Mechanistically, pyroptotic cell death occurs upon cleavage of Gasdermin-D (GSDMD), a pore-forming effector protein that is cleaved by caspase-1 and caspase-4/11^21–23^. GSDMD is composed of an effector N-terminal region and a C-terminal region with inhibitory functions. Upon inflammasome activation, caspase-1 and/or caspase-11 cleave GSDMD, releasing the N-terminal fragment that is the effector domain. The N-terminal portion of the protein oligomerizes in the plasma membrane, forming ring-shaped pores that cause cell death due to the release of intracellular content and osmotic imbalance (reviewed in ref. ^24^). In addition, the pores formed by GSDMD work as channels for releasing IL-1β, which can occur even in viable cells^25^. Such characteristics make GSDMD of pivotal importance in the induction of protective immune responses against intracellular pathogens^24^.

Although many studies performed by different groups have demonstrated the critical role of the inflammasomes in the host response to *Leishmania*, the precise molecular mechanisms that lead to inflammasome activation remain unclear. It was previously shown that host signaling pathways such as Dectin-1/Syk Kinase and ROS production generated during parasite phagocytosis account for NLRP3 activation^18^. In addition, the efflux of K^+^, which is well reported to be essential for NLRP3 activation in response to many stimuli, is indeed required for inflammasome activation in response to *Leishmania* in mouse and human cells^15,17,26^. In this context, the requirement of K^+^ efflux for NLRP3 activation in *Leishmania*-infected cells is paradoxical because *Leishmania* parasites actively prevent plasma membrane damage to avoid host cell death, as extensively reported^27–30^. In this study, we evaluate the role of the GSDMD in *Leishmania* infection. We demonstrate that GSDMD is critical in the immunity to Leishmaniasis. Using functional analysis of plasma membrane permeability, live-cell visualization, and immunofluorescence in infected macrophages, we found that GSDMD activation occurs transiently and in the first hours of infection, triggering the K^+^ efflux and the noncanonical activation of the NLRP3 but not cell death. This study accounts for our understanding of the pathogenesis and host response to *Leishmania* and also opens the possibility of investigating a new pathway that has the potential to be used for the design of new drug targets in the fight against Leishmaniasis.

## Results

### *Leishmania* induces GSDMD activation but not cell death

**Fig. 1:**
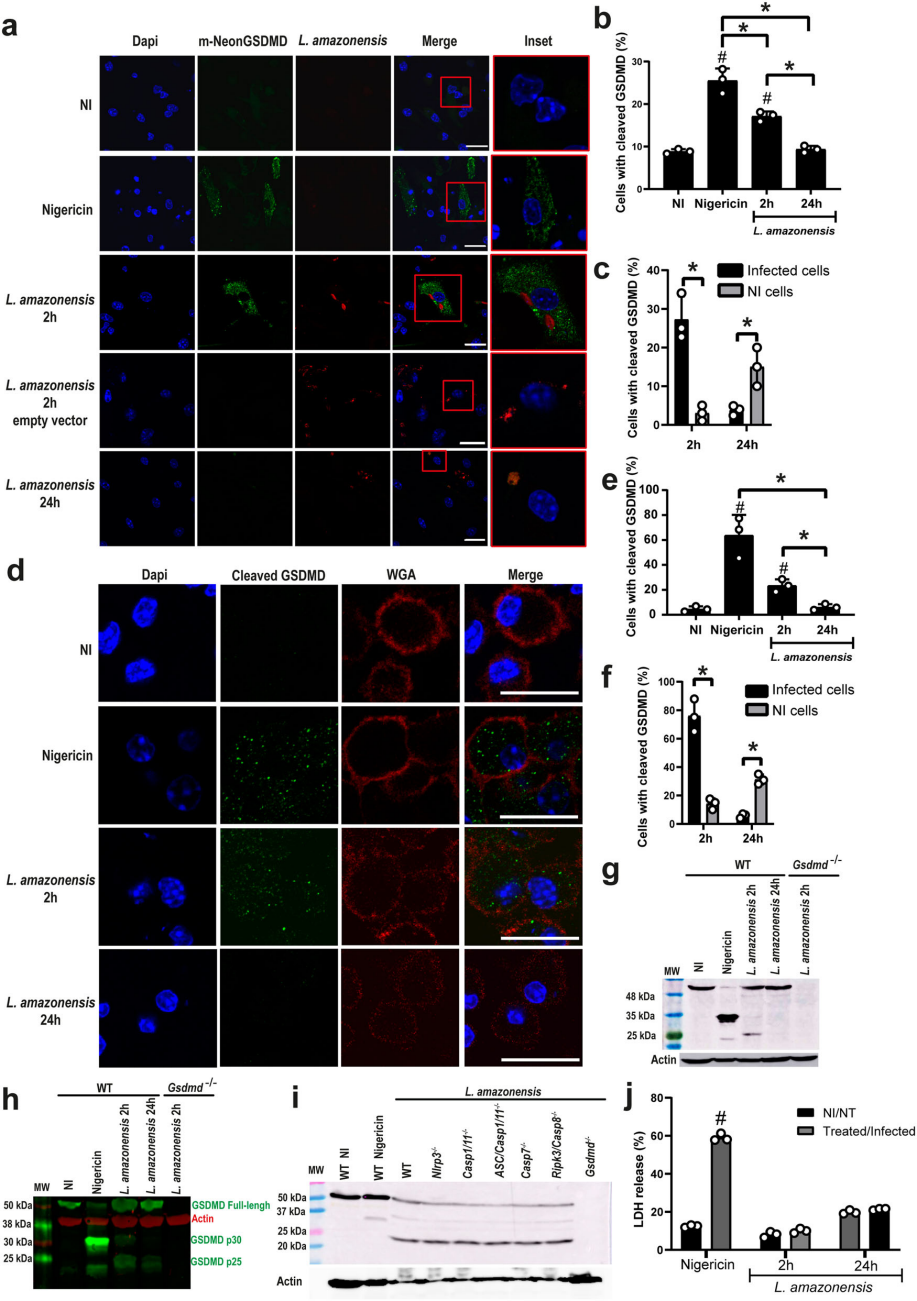
*Leishmania* induces a noncanonical GSDMD cleavage in macrophages. **a**–**c** BMDMs from *Gsdmd*^*–/–*^ mice were transduced with a lentivirus encoding GSDMD-mNeon. **a** Representative images of BMDMs expressing GSDMD-mNeon, pretreated for 4 h with LPS (100 ng/mL) and infected with *L. amazonensis-*RFP (red); MOI 3. The cell nucleus was marked with Dapi (blue). Cleaved GSDMD (N-terminal) in green. Scale bar 20 µm. **b** Percentage of transduced BMDMs containing cleaved GSDMD after 4 and 24 h infection with *L. amazonensis*. Nigericin was used as a positive control. **c** Percentage of infected versus non-infected BMDMs containing cleaved GSDMD (N-terminal). A total of 100 cells in each triplicate well were analyzed. **d**–**f** BMDMs from C57BL/6 mice were pretreated for 4 h with LPS (100 ng/mL) and infected with an MOI 5. **d** Representative images of BMDMs stained with anti-C-terminal GSDMD showing cleavage of endogenous GSDMD (green). Plasma membrane was marked with WGA (red). Scale bar 20 µm. **e** Percentage of BMDMs with endogenous GSDMD cleavage (C-terminal) after 2 and 24 hs infection. **f** Percentage of infected and non-infected BMDMs with endogenous GSDMD cleavage (C-terminal). A total of 100 cells in each triplicate well were analyzed. **g**–**h** Lysates from C57BL/6 and *Gsdmd*^*–/–*^ BMDMs pretreated for 4h with LPS (100 ng/mL) and infected for 2 or 24 h were assessed for GSDMD cleavage by western blot. Show are the same experiment stained with conventional chemiluminescence (**g**) or near-infrared fluorescence (**h**). **i** Lysates from WT, *Nlrp3*^*–/–*^, *Casp1/11*^*–/–*^, *Asc/Casp1/11*^*–/–*^, *Casp7*^*–/–*^, *Ripk3/Casp8*^*–/–*^, and *Gsdmd*^*–/–*^ BMDMs pretreated for 4 h with LPS (100 ng/mL) and infected for 2 h were assessed for GSDMD cleavage by western blot. MW, Molecular Weight. **j** LDH release was assessed 2 h and 24 h after infection or 2 h after treatment with Nigericin. NI non-infected, NT non-treated. Data are presented as mean values ±SD of triplicate wells. ^#^*P* < 0.05 compared with NI/NT cells; *P < 0.05 comparing the indicated groups, as determined by two-way ANOVA. Shown is one representative experiment of five independent experiments performed. Source data are provided as a Source Data file.

To investigate GSDMD activation and pore formation in macrophages infected with *Leishmania*, we performed a previously described protocol using a GSDMD-mNeon-tagged on its N-terminal region that allows visualization of cleaved GSDMD via the mNeon tag fluorescence^31^. Bone marrow-derived macrophages (BMDMs) transduced with retrovirus encoding mNeon-tagged GSDMD were infected with stationary-phase *L.* amazonensis-expressing RFP for 2, and 24 h, and GSDMD cleavage was accessed by immunofluorescence. We detected GSDMD cleavage after 2 h infection, but after 24 h infection, we found a significant reduction in GSDMD cleavage (Fig. 1a, b). Nigericin, a bacterial toxin known to trigger inflammasome activation, was used as a positive control (Fig. 1a, b). We scored the GSDMD cleavage in infected versus non-infected macrophages and found that most of the cells with cleaved GSDMD at 2 h are infected (Fig. 1c). By contrast, at 24 h infection, only the non-infected cells contain active GSDMD, suggesting that *Leishmania* inhibits the canonical cleavage of GSDMD latter after infection (Fig. 1c). We next tested the cleavage of endogenous GSDMD by staining the C-terminal fragment of GSDMD. We found that endogenous GSDMD is cleaved in response to *Leishmania amazonensis* after 2 h infection but not after 24 h (Fig. 1d, e). Analysis of C-terminal GSDMD in infected versus non-infected macrophages indicates that the majority of the cells with cleaved C-terminal GSDMD at 2 h are infected as opposed to 24 h infection (Fig. 1f). To further assess GSDMD cleavage in response to *Leishmania* infection, we infected BMDMs for 2 and 24 h and evaluated GSDMD processing by western blot. Interestingly, we found that different from the canonical 35 kDa product induced by Nigericin, *Leishmania* infection induces the production of a noncanonical 25 kDa fragment (Fig. 1g, h). We further investigated the participation of caspases in the production of this noncanonical GSDMD cleavage and found that production of the 25 kDa fragment occurs in *Nlrp3*^–*/*–^, *Casp1/11*^–/–^, *Asc/Casp1/11*^*–/–*^*, Casp7*^*–/–*^*, Casp8/Rip3*^*–/–*^ macrophages (Fig. 1i). These data suggest that there is a redundancy of proteases involved in this alternative GSDMD cleavage, or a yet unidentified protease is involved in this noncanonical cleavage of GSDMD in response to *Leishmania* infection. Finally, we assessed LDH release in response to *Leishmani*a infection as a measurement of host cell lysis and pyroptosis and found no LDH release in BMDMs infected with *Leishmania* for 2 or 24 h (Fig. 1j).

It was previously described that ESCRT-dependent membrane repair negatively regulates pyroptosis downstream of GSDMD activation^32,33^. Thus, to evaluate if *Leishmania* triggers ESCRT machinery to inhibit pyroptosis, we treated BMDMs with a calcium chelator BAPTA, which is known to inhibit ESCRT activation^33^. If this hypothesis was correct, we expected ESCRT inhibition to result in cell death and LDH release, but this was not observed. No LDH was detected in Leishmania-infected cells in the presence of BAPTA. In contrast, BAPTA was efficient in increasing the LDH release in response to Nigericin (Supplementary Fig. 1). These results do not support the role of ESCRT machinery in the inhibition of lytic cell death induced by *Leishmania* and confirm that *Leishmania* infection inhibits pyroptosis despite the transient activation of GSDMD in the infected macrophages.

### *Leishmania* induces GSDMD transient cell permeabilization

**Fig. 2:**
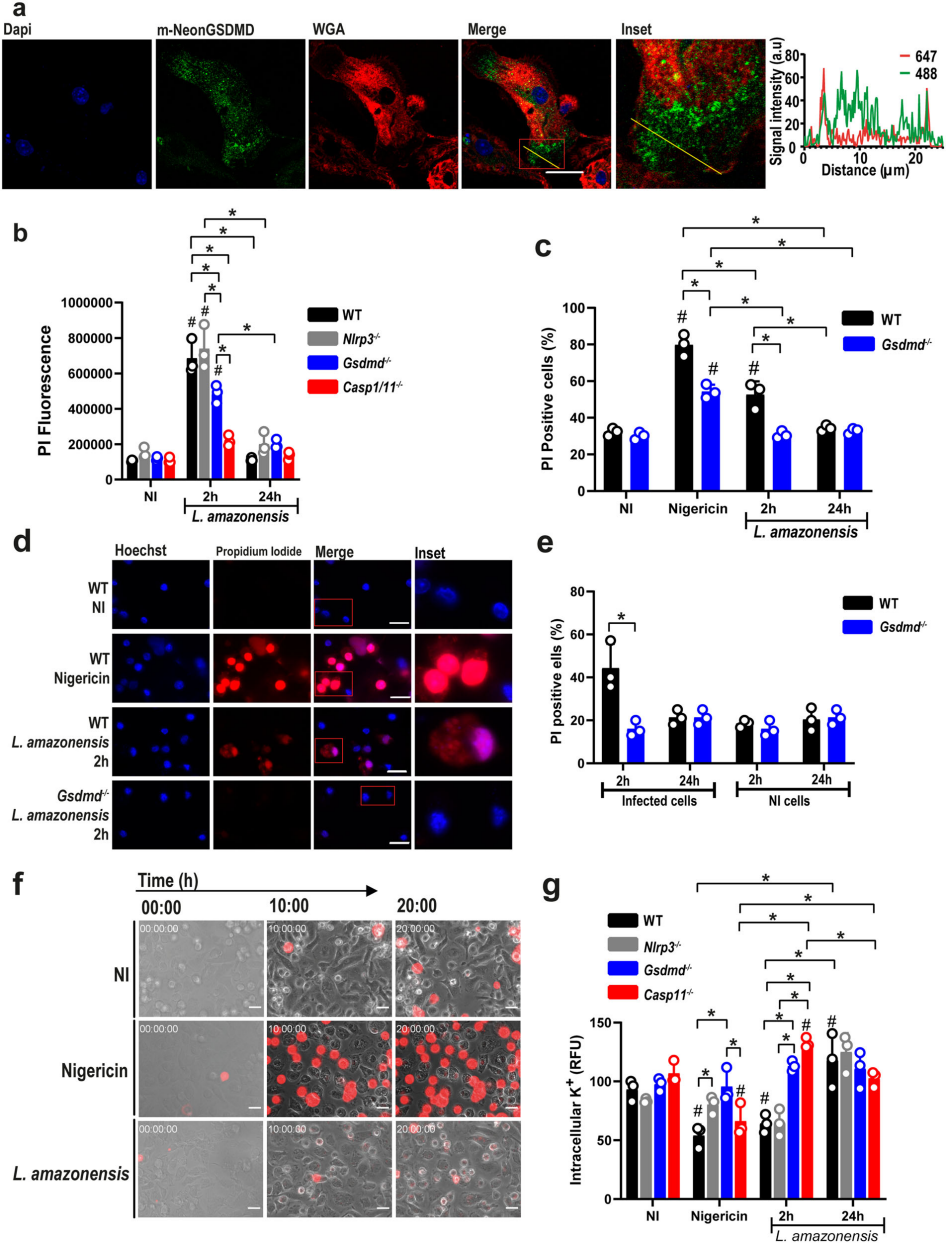
GSDMD induces transient macrophage permeabilization upon infection. **a** BMDMs from *Gsdmd*^*–/–*^ mice were transduced with a lentivirus encoding GSDMD-mNeon. Representative images of BMDMs expressing GSDMD-mNeon (green), pretreated for 4 h with LPS (100 ng/mL) and infected with *L. amazonensis;* MOI 3. Cell nucleus was marked with Dapi (blue), plasma membrane was marked with WGA (red). Yellow line indicates the scan analysis in the graph. Scale bar 20 µm. **b** BMDMs from WT, *Nlrp3*^*–/–*^, *Gsdmd*^*–/–*^ and C*asp1/11*^*–/–*^ mice were pretreated for 4 h with LPS (100 ng/mL) and left non-infected (NI) or infected (MOI 10) for 4 h or 24 h. Cell permeabilization was assessed by flow cytometry upon propidium iodide (PI) staining. **c**–**e** BMDMs were pretreated for 4 h with LPS (100 ng/mL) and infected at an MOI 10 for 2 h or 24 h; the cultures were stained with PI (red) and Hoechst (blue) and assayed for pore formation by microscopy. **d** Representative images of pore formation. A total of 100 cells in each triplicate well were analyzed. Scale bar 20 µm. **f** Live-cell visualization of BMDMs from WT mice pretreated for 4 h with LPS (100 ng/mL) and with PI in the culture medium. Images (0, 10, and 20 h post infection/treatment) were acquired during a video (see Supplementary Movies 1–3). Scale bar 20 µm. **g** BMDMs were pretreated for 4 h with LPS (100 ng/mL) and left non-infected (NI), treated with nigericin (10 µM), or infected for 2 h and 24 h (MOI 10) to assess intracellular K^+^. Each bar represents the percentage of APG-2 fluorescence intensity in relation to the average fluorescence of non-infected (NI) cells. RFU, relative fluorescence units. Data are presented as mean values ± SD of triplicate wells. ^#^*P* < 0.05 compared to NI; *P < 0.05 comparing the indicated groups, as determined by two-way ANOVA. Shown is one representative experiment of five independent experiments performed. Source data are provided as a Source Data file.

Even though LDH levels did not increase with GSDMD activation in response to *Leishmania* infection, we asked whether cleaved GSDMD was located in the plasma membrane. We performed immunofluorescence assays using GSDMD-mNeon and stained macrophage membranes with wheat germ agglutinin (WGA). A Z-stack was made with slices of 0.32 µm, and a single slice located in the middle of the cell was used to assess co-localization at 2 h p.i. We found active GSDMD diffusely distributed through the cell and also in the plasma membrane, which was confirmed by the yellow line (Fig. 2a). To investigate whether transient cell permeabilization was occurring, we infected BMDMs from wild type (WT), *Nlrp3*^*–/–*^, *Casp1/11*^*–/–*^, and *Gsdmd*^*–/–*^ mice with *L. amazonensis* and measured propidium iodide (PI) uptake by flow cytometer (gating strategy are represented in Supplementary Fig. 2). In WT macrophages, we observed PI incorporation after 4 h infection and this early cell permeabilization was GSDMD and Caspase-1/11-dependent and NLRP3 independent, suggesting that GSDMD cleavage occurs upstream to the NLRP3 inflammasome activation (Fig. 2b). This data is consistent with the previously reported role of Caspase-11 in the noncanonical activation of the NLRP3 during *Leishmania* infection^13,14,34^. It is worth noticing that PI incorporation at 24 h after infection is similar to that observed in non-infected cells (NI), suggesting a transient permeabilization of the cells (Fig. 2b). We also assessed pore formation by microscopy scoring the PI^+^ cells as described^35^. BMDMs were stained with Hoechst, and PI incorporation was assessed under a fluorescence microscope in WT and *Gsdmd*^*–/–*^ BMDMs infected with *L. amazonensis* for 2 h and 24 h. After 2 h of infection, we observed a weak PI staining in WT but not in *Gsdmd*^*–/–*^ BMDMs. However, no PI incorporation was detected after 24 h infection (Fig. 2c, d). Nigericin was used as a positive control for inflammasome activation and pore formation. Interestingly, only infected cells were PI^+^ after 2 h infection, suggesting that *Leishmania* internalization in macrophages is required for inflammasome activation and the transient GSDMD-mediated pore formation (Fig. 2e). We also performed these experiments using metacyclic promastigotes of *L. amazonensis* and PAM3Cys for primming (because LPS can activate Caspase-11 directly) and obtained similar results (Supplementary Fig. 3a–c).

To rule out the possibility that the lack of PI uptake after 24 h was due to cell death in the first hours of infection, we performed a live-cell visualization for 20 h with cells infected with *L. amazonensis*, stimulated with LPS or stimulated with LPS + nigericin. After 20 h, a stronger PI staining was observed in Nigericin-stimulated cells compared to *Leishmania*-infected cells. In the *L*. *amazonensis-*infected cells, we did not observe significant cell death when compared with non-infected cultures. However, we detected a weak PI staining in *Leishmania-*infected cultures (Fig. 2f and Supplementary Movies 1–3), consistent with the FACS and microscopy PI staining data.

The imbalance of ionic cell homeostasis occurs when GSDMD-induced pores are formed, allowing ions such as potassium to escape and sodium and water to enter, which leads to cell swelling and rupture of the plasma membrane^36^. Potassium efflux is an essential activator of the NLRP3 inflammasome, and it has been previously shown that it is important for the NLRP3 inflammasome activation in *L. amazonensis* infection^17^. To assess whether the potassium efflux is GSDMD-dependent, BMDMs from WT, *Nlrp3*^*–/–*^, *Casp11*^*–/–*^, and *Gsdmd*^*–/–*^ mice were infected with *L. amazonensis*, and the intracellular potassium was stained using an APG-2 probe at different time points. In WT cells, we observed a significant potassium efflux early after infection (2 h p.i.) but not after 24 h infection (Fig. 2g). Confirming the PI uptake data, potassium efflux occurred in WT and *Nlrp3*^*–/–*^ cells but not in *Gsdmd*^*–/–*^ and *Casp11*^*–/–*^. Our results suggest that *L. amazonensis* infection triggers GSDMD activation, leading to transient pore formation and potassium efflux, but it does not lead to cell death.

### GSDMD is important for NLRP3 activation in infected BMDMs

**Fig. 3:**
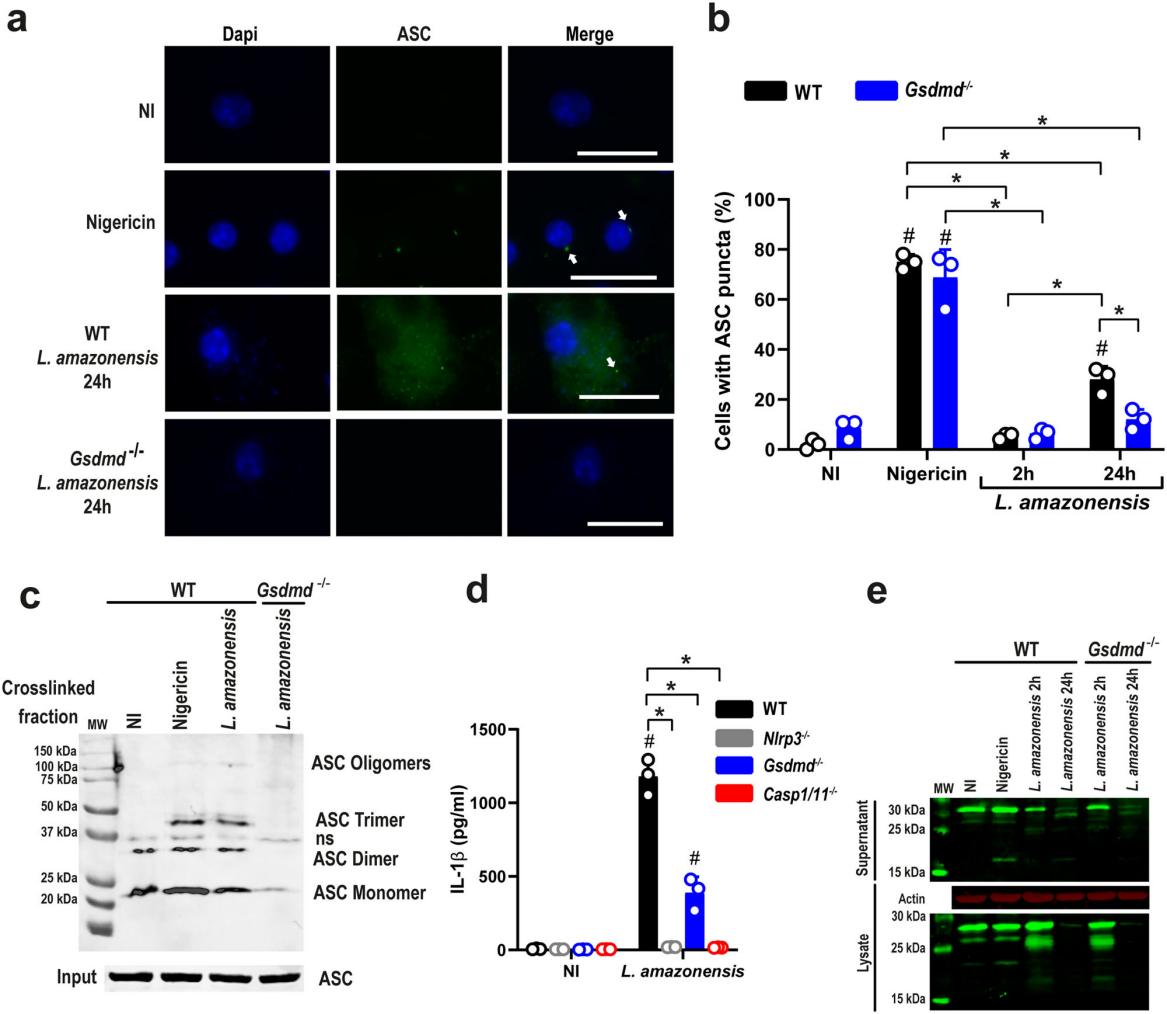
GSDMD is important for inflammasome activation in *Leishmania*-infected macrophages. **a**, **b** Bone marrow-derived macrophages (BMDMs) generated from C57BL/6 (WT) and *Gsdmd*^*–/–*^ mice were pretreated for 4 h with LPS (100 ng/mL) and infected with *L. amazonensis* at an MOI 10 for 2 h or 24 h. The cultures were stained with anti-ASC (green, indicated by arrows), and cell nuclei were stained with DAPI (blue). **a** Representative images of ASC puncta formation in response to *L. amazonensis* infection. Images were acquired by fluorescence microscopy with a ×100 oil immersion objective and analyzed using ImageJ software. Scale bar 20 µm. **b** The percentage of ASC puncta in BMDMs infected by *L. amazonensis* for 2 or 24 hs. Nigericin was used as a positive control. A total of 100 cells in each triplicate well were analyzed. **c** Lysates from C57BL/6 (WT) and *Gsdmd*^−/−^ macrophages were pretreated for 4 h with LPS (100 ng/mL) and infected with *L. amazonensis* for 24 h assessed for ASC oligomerization by western blot. ASC monomers, dimers, trimers, and oligomers are indicated in the figure. ns non-specific band. **d** BMDMs from WT, *Gsdmd*^*–/–*^, *Nlrp3*^*–/–*^, and *Casp1/11*^*–/–*^ mice were treated with LPS (100 ng/mL) for 4 h and infected with *L. amazonensis* at an MOI of 10 for 24 h. IL-1β production was measured by ELISA. **e** Western blot analyses of lysate and supernatant of WT and *Gsdmd*^*–/–*^ BMDMs pretreated for 4 h with LPS (100 ng/mL) and were left non-infected (NI) or infected by *Leishmania* at an MOI 10 for 2 h or 24 h. The unprocessed (31 kDa) and processed (17 kDa) IL-1β are indicated in the figure. MW molecular weight. Data are presented as mean values ± SD of triplicate wells. ^#^*P* < 0.05 compared with NI cells; *P < 0.05 comparing the indicated groups, as determined by two-way ANOVA. Shown is one representative experiment of five independent experiments performed. Source data are provided as a Source Data file.

Our data indicating that NLRP3 but not Caspase-11 is dispensable for GSDMD-mediated K^+^ efflux suggests that GSDMD is upstream of NLRP3 activation. To further test this hypothesis, we assessed the formation of ASC puncta (or specks) that has been previously shown to occur in response to *L. amazonensis* infection in BMDMs^15,17,37^. To test the effect of GSDMD in ASC puncta formation, we infected WT and *Gsdmd*^*–/–*^ BMDMs with *L. amazonensis* and evaluated puncta formation by immunofluorescence after 24 h of infection. We found that ASC puncta were observed in higher levels in WT compared with *Gsdmd*^*–/–*^ BMDM (Fig. 3a, b). We next tested the formation of ASC oligomers by western blot. We found that after 2 h infection, *Leishmania* induces ASC oligomerization, and this process was GSDMD-dependent (Fig. 3c). We also tested IL-1β secretion upon *Leishmania* infection, as this process has been previously shown to be dependent on the NLRP3 inflammasome^12,16,17^. To determine whether GSDMD is important for IL-1β release, we infected WT and *Gsdmd*^*–/–*^ BMDMs with *L. amazonensis* and measured IL-1β in tissue culture supernatants by ELISA. Our data show that efficient IL-1β production in response to *L. amazonensis* requires GSDMD as well as NLRP3 and caspase-1/11 (Fig. 3d). This result is consistent with data previously described, indicating a role for the noncanonical pathway in the activation of NLRP3 in *Leishmania*^13,14^. Western blot analysis of the supernatant and lysate from WT and *Gsdmd*^*–/–*^ BMDM infected with *L. amazonensis* confirms the role of GSDMD in IL-1β maturation and secretion (Fig. 3e). Next, we tested the effect of GSDMD in inflammasome activation using additional forms of *Leishmania* and PAM3Cys for primming, because cytoplasmic LPS activates caspase-11 and triggers the noncanonical activation of the NLRP3 inflammasome^38,39^. Using amastigote forms of the parasite, which express significantly less LPG than promastigotes (and consequently induce less inflammasome activation as compared to promastigotes^14^), we found that GSDMD is also important for inflammasome activation induced by *L. amazonensis* axenic amastigotes, as measured by IL-1β production (Supplementary Fig. 4a). We also found that GSDMD is important for inflammasome activation in response to metacyclic promastigotes of *L. amazonensis* (Supplementary Fig. 4b). Finally, we tested the effect of priming for the noncanonical cleavage of GSDMD induced by *L. amazonensis*. We found that although priming is not required for producing a 25 kDa fragment of GSDMD, pretreatment of cells with PAM3Cys significantly increased the GSDMD cleavage (Supplementary Fig. 4c). Together, these results show that GSDMD is important for ASC oligomerization/puncta formation and IL-1β release upon *L. amazonensis* infection.

### GSDMD promotes *Leishmania* growth restriction in BMDMs

**Fig. 4:**
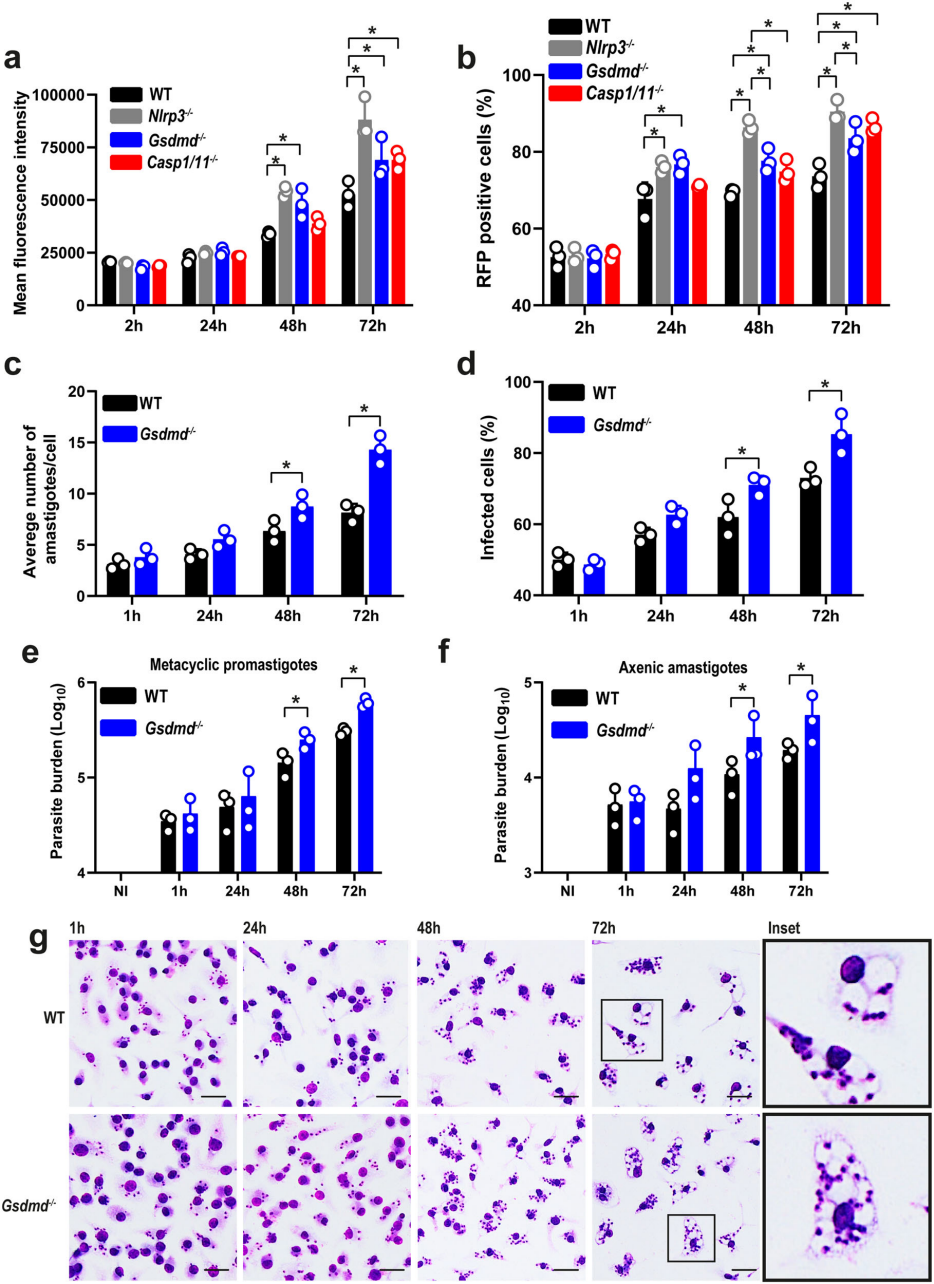
GSDMD accounts for the restriction of *L. amazonensis* infection in bone marrow-derived macrophages (BMDMs). **a**, **b** Flow cytometry analysis of C57BL/6 (WT), *Nlrp3*^*–/–*^, *Gsdmd*^*–/–*^, and *Casp1/11*^*–/–*^ BMDMs infected with stationary-phase promastigotes of *L. amazonensis* constitutively expressing RFP. Cells were infected at an MOI 5 for 2 h, washed, and incubated for 24, 48, and 72 h. **a** Mean fluorescence intensity of *L. amazonensis-*expressing RFP. (**b**) The percentage of RFP-positive BMDMs. **c**, **d** Giemsa staining of C57BL/6 (WT) and *Gsdmd*^*–/–*^ BMDMs infected with metacyclic promastigotes of *L. amazonensis* at an MOI 5. Cultures were infected for 1 h, washed, and incubated for 24, 48, and 72 h. **c** The average number of amastigotes per BMDMs. **d** The percentage of infected BMDMs. A total of 100 cells in each triplicate well were analyzed. **e**, **f** Parasite quantification by real-time PCR in C57BL/6 (WT) and *Gsdmd*^*–/–*^ BMDMs infected with metacyclic promastigotes (**e**) and axenic amastigotes (**f**). **g** Representative images of Giemsa-stained cultures show intracellular amastigotes in BMDMs, scale bar 50 µm. Data are presented as mean values ± SD of triplicate wells. *P < 0.05 comparing the indicated groups, as determined by two-way ANOVA. Shown is one representative experiment of five independent experiments performed. Source data are provided as a Source Data file.

To evaluate whether GSDMD influences *Leishmania* replication in BMDMs, we infected WT, *Nlrp3*^*–/–*^, *Gsdmd*^*–/–*^, and *Casp1/11*^*–/–*^ BMDMs with metacyclic promastigotes of *L. amazonensis*-expressing RFP and measured parasite replication by FACS. The internalization of the parasites after 2 h of infection was not affected by the deficiency of GSDMD, NLRP3, or Caspase-1/11, but macrophages lacking any of these genes were more susceptible to intracellular replication of the parasite as observed after 48 h and 72 h of infection (Fig. 4a, b). To confirm these data, we also infected WT and *Gsdmd*^*–/–*^ BMDMs with metacyclic forms of *L. amazonensis* and measured parasite load by Giemsa staining. We found an increased parasite load in *Gsdmd*^*–/–*^ compared with WT BMDM (Fig. 4c). The increased susceptibility of *Gsdmd*-deficient cells was also observed when we scored the percentage of infected cells and we can observe that at one hour of infection, internalization of the parasites was similar for both groups. After 48 and 72 h, we detected an increased proportion of infected cells in *Gsdmd*^*–/–*^ BMDMs (Fig. 4d). We also measured parasite load in the cultures using qPCR and also found an increased number of parasites in *Gsdmd*^*–/–*^ BMDMs (Fig. 4e). This was observed both in infections with metacyclic promastigotes and axenic amastigotes (Fig. 4e, f). Representative images of Giemsa-stained cultures of BMDMs infected with axenic amastigotes for 1, 24, 48, and 72 h post infection are shown in Fig. 4g.

**Fig. 5:**
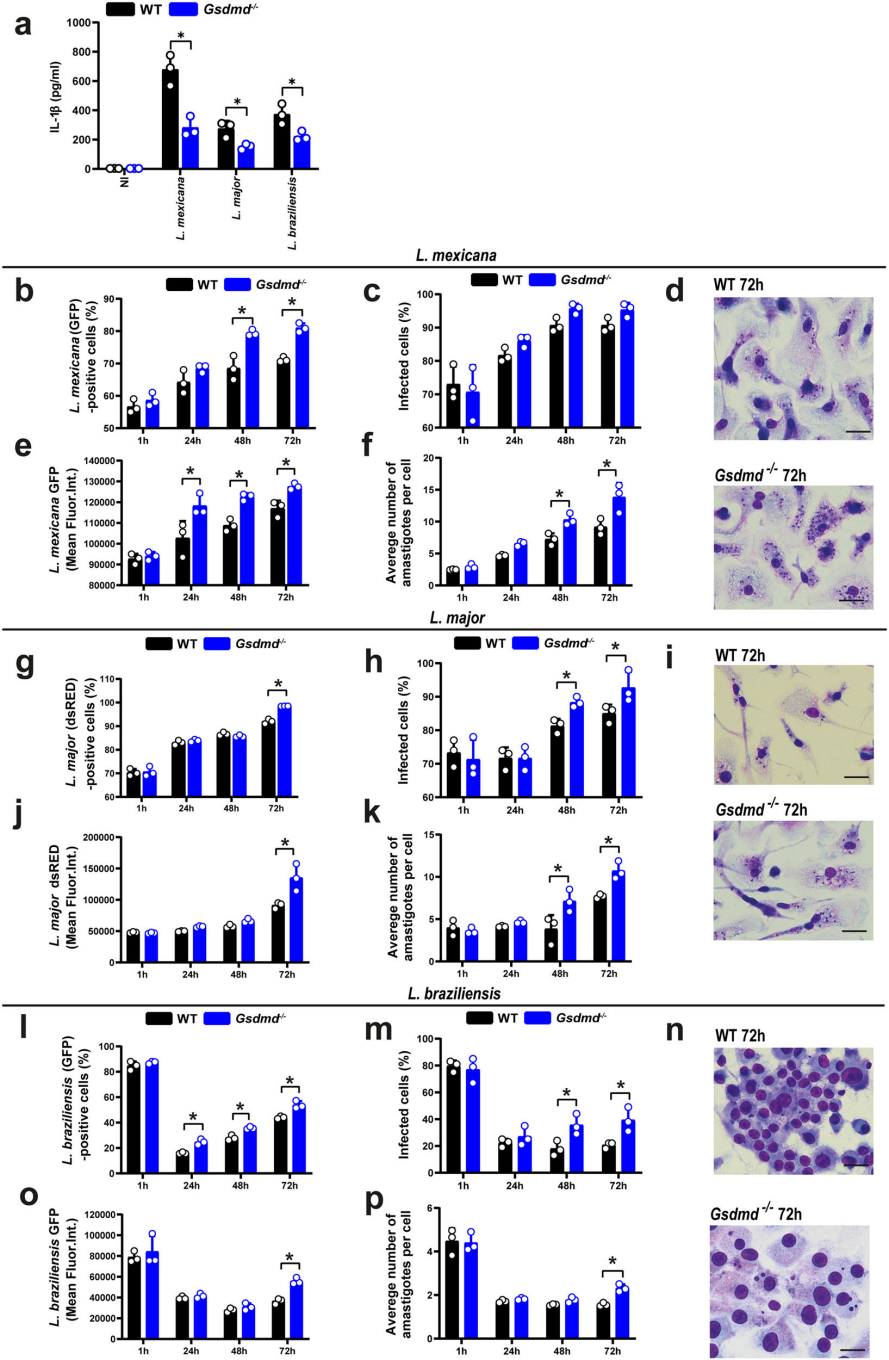
GSDMD is important for the restriction of *L. mexicana, L. major*, and *L. braziliensis* in macrophages. **a** Bone marrow-derived macrophages (BMDMs) from C57BL/6 (WT) and *Gsdmd*^*–/–*^ mice were pretreated for 4 h with LPS (100 ng/mL) and infected with stationary-phase *L. mexicana, L. major, L. braziliensis* MOI 10 for 24 h. IL-1β released in BMDM supernatant was measured by ELISA. **b**–**n** BMDMs from WT and *Gsdmd*^*–/–*^ mice were infected with stationary-phase parasites at an MOI 5 for 2 h, washed, and incubated for 1, 24, 48, and 72 h. Parasites used were: *L. mexicana* constitutively expressing GFP (**b**–**f**), *L. major* constitutively expressing dsRED (**g**–**k**), and *L. braziliensis* constitutively expressing GFP (**l**–**p**). **b**, **g**, **l** The percentage of infected cells was estimated by flow cytometry. **e**, **j**, **o** The mean fluorescence intensity of intracellular parasites was estimated by flow cytometry. **c**, **h**, **m** The percentage of infected cells was scored in Giemsa-stained cells. **f**, **k**, **p** The average number of amastigotes per cell was scored in the Giemsa-stained cells. A total of 100 cells in each triplicate well were analyzed. **d**, **i**, **n** Representative images of Giemsa-stained cultures showing intracellular amastigotes. Scale bar 50 µm. Data are presented as mean values ± SD of triplicate wells. *P < 0.05 comparing the indicated groups, as determined by two-way ANOVA. Shown is one representative experiment of three independent experiments performed. Source data are provided as a Source Data file.

Next, we investigated if the GSDMD-mediated inflammasome activation and restriction of *L. amazonensis* replication occurred with other species of *Leishmania*. To test this, WT and *Gsdmd*^*–/–*^ BMDMs were infected with *L. mexicana, L. major*, and *L. braziliensis* for 24 h, and the IL-1β levels were measured in the tissue culture supernatants as a readout for inflammasome activation. Our data show that GSDMD is required for efficient IL-1β secretion in response to all species tested, indicating that GSDMD-mediated inflammasome activation was a general mechanism operating in response to *Leishmania* spp. (Fig. 5a). To test whether GSDMD was important for restricting intracellular replication of *Leishmania* species, we infected BMDMs from WT and *Gsdmd*^*–/–*^ mice with stationary-phase promastigotes of *L. amazonensis, L. mexicana* expressing GFP*, L. major* expressing dsRED and *L. braziliensis* expressing GFP and measured parasite replication by FACS. The gating strategy are shown in Supplementary Fig. 2b. The internalization of the parasites after 4 h of infection was not affected by the deficiency of GSDMD, but BMDMs were more susceptible to intracellular replication of the parasite as observed after 48 h and 72 h of infection (Fig. 5b, e, g, j, l, o). Assessment of intracellular parasite loads also indicates an important role of GSDMD in the restriction of intracellular replication of *Leishmania* species in macrophages. This was evident in measuring parasite loads by FACS (Fig. 5c, f, h, k, m, p) and Giemsa staining (Fig. 5d, i, n).

### GSDMD promotes restriction of *Leishmania* infection in vivo

**Fig. 6:**
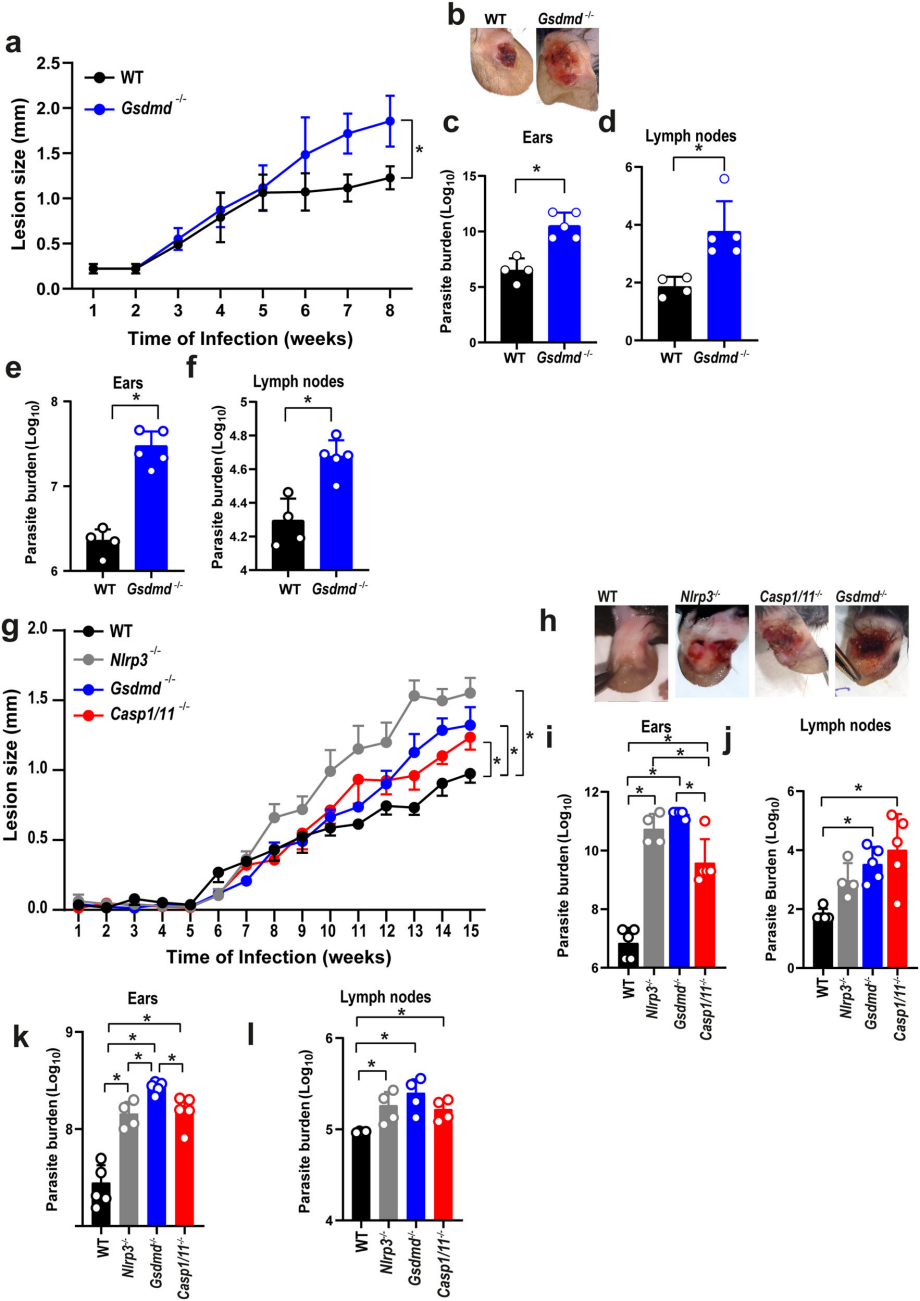
GSDMD is important for the restriction of *L. amazonensis* infection in vivo. **a**–**f** C57BL/6 (WT) and *Gsdmd*^*–/–*^ mice were infected with 10^6^ stationary-phase *L. amazonensis* promastigotes in the ear, and the ear thicknesses were followed for 8 weeks. **a** Lesion development; **b** images of infected ears; **c**, **d** limiting dilution analysis of parasite burden in the infected ears (**c**), and draining lymph nodes (**d**); **e**, **f** parasite quantification by real-time PCR in the infected ears (**e**), and draining lymph nodes (**f**) at 8 weeks of infection. Each dot in the bar graphics represents the value obtained from an individual mouse. Data are presented as mean values ± SD. #*P* < 0.05 compared with WT mice; *P < 0.05 comparing the indicated groups, as determined by the Student T test two-sided. **g**, **l** C57BL/6 (WT), *Nlrp3*^*–/–*^, *Casp1/11*^*–/–*^, and *Gsdmd*^*–/–*^ mice were infected with 10^3^ metacyclic *L. amazonensis* promastigotes in the ear, and the ear thicknesses were followed for 15 weeks. **g** Lesion development; (**h**) images of infected ears; **i**, **j** limiting dilution analysis of parasite burden in the infected ear (**i**) and draining lymph nodes (**j**); **k**, **l** parasite quantification by real-time PCR in the infected ears (**k**), and draining lymph nodes (**l**) at 15 weeks of infection. Each dot in the bar graphics represents the value obtained from an individual mouse. Data are presented as mean values ± SD. *P < 0.05 comparing the indicated groups, as determined by two-way ANOVA. Shown is one representative experiment of five independent experiments performed. Source data are provided as a Source Data file.

**Fig. 7:**
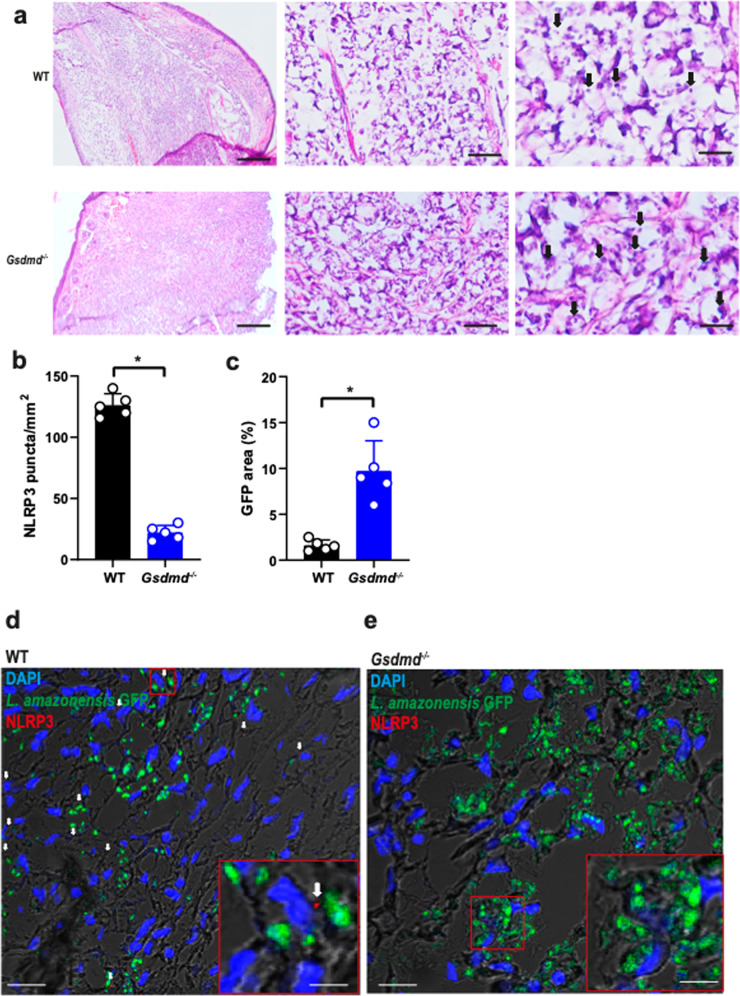
GSDMD is important for the restriction of *L. amazonensis* infection and NLRP3 inflammasome activation in vivo. C57BL/6 (WT) and *Gsdmd*^*–/–*^ mice (*n* = 5 mice per group) were infected with 10^6^ stationary-phase promastigotes of *L. amazonensis*-expressing GFP. (**a**) Hematoxylin and Eosin staining of the infected ear indicates the amastigotes (arrows). Scale bars, 500, 50, and 20 µm (from left to right). **b**–**e** Multiphoton microscopy of infected ears stained with anti-NLRP3 for quantification of NLRP3 puncta formation (**b**) and percentage of GFP area (**c**). Representative images of WT (**d**) and *Gsdmd*^*–/–*^ mice (**e**) showing NLRP3 puncta (in red, indicated by a white arrow). GFP-expressing *Leishmania* is shown in green, and DAPI stains cell nuclei (blue). Scale bar 40 µm. Insets indicate a higher magnification of a region indicated (red rectangle, scale bar 20 µm). Each dot in the figure represents the value obtained from an individual mouse. Data are presented as mean values ± SD. *P < 0.05 comparing the indicated groups, as determined by the Student *T* test two-sided. Source data are provided as a Source Data file.

To assess the physiological relevance of GSDMD in vivo, we performed infections in mouse ears as previously described^40^. Initially, we used 10^6^ stationary-phase promastigotes of *L. amazonensis* to infect WT and *Gsdmd*^*–/–*^ mice for 8 weeks. We found a significant increase in the lesion size (Fig. 6a, b) and parasite burden as measured by limiting dilution assay in the ears and draining lymph nodes in *Gsdmd*^*–/–*^ mice compared to WT (Fig. 6c, d). We also measured parasite burden in the ears and lymph nodes of mice using real-time PCR and found higher parasite loads in *Gsdmd*^*–/–*^ compared to WT mice (Fig. 6e, f). Next, we compared the infections of WT, *Nlrp3*^*–/–*^, *Gsdmd*^*–/–*^, and *Casp1/11*^*–/–*^ mice using metacyclic promastigotes. Mice were intradermally infected in the ear with 10^3^ metacyclic promastigotes of *L. amazonensis*, and the size of the lesion were measured during 15 weeks. We found a significant increase in lesion size and skin necrosis in the ears of *Gsdmd*^*–/–*^, *Nlrp3*^*–/–*^, and *Casp1/11*^*–/–*^ mice (Fig. 6g, h). The mice were euthanatized in the 15^th^ week, and parasite burdens were measured in the ears and draining lymph nodes. At 15 weeks post infection, we detected increased parasite loads in the ears of *Gsdmd*^*–/–*^, *Nlrp3*^*–/–*^, and *Casp1/11*^*–/–*^ as compared to WT mice (Fig. 6i, j). We also quantified the parasite loads in the ears of mice by real-time PCR and we confirmed increased parasite loads in the ears of *Gsdmd*^*–/–*^, *Nlrp3*^*–/–*^, and *Casp1/11*^*–/–*^ as compared to WT mice (Fig. 6k, l). The increased susceptibility of the *Gsdmd*^*–/–*^ mice to *L. amazonensis* infection prompted us to assess further the parasite loads and inflammasome activation in the tissues of infected mice. Initially, we performed H&E staining of histological sections of mice ears infected for 15th weeks, revealing a higher number of parasites in the *Gsdmd*^*–/–*^ (Fig. 7a). We stained sections of infected ears with anti-NLRP3 to assess puncta formation (indicating inflammasome activation) in the infected tissues. We found a significant reduction in inflammasome activation in the ears of *Gsdmd*^*–/–*^ mice (Fig. 7b). In this experiment, we infected mice with GFP-expressing *L. amazonensis* to allow GFP quantification in tissue sections, assessing parasite burden in the tissues. We found increased parasite loads in the ears of *Gsdmd*^*–/–*^ mice (Fig. 7c), consistent with the data generated by limiting dilution assay and real-time PCR (Fig. 6). Representative images of infected tissues with active inflammasomes and *Leishmania* amastigotes are shown (Fig. 7d, e). This data confirms the increased susceptibility of *Gsdmd*^*–/–*^ mice and highlight the important role of GSDMD for inflammasome activation. Importantly, these data show, for the first time, inflammasome activation in mouse tissues in vivo.

To gain insight into the immune response profile that may confer greater susceptibility to *Leishmania* infection in vivo in the *Gsdmd*^−/−^ mice, we measured inflammatory cytokines by ELISA or CBA (Supplementary Fig. 5a–g) and qPCR (Supplementary Fig. 5h–x) in the ear and lymph node of mouse infected intradermally in the ear with 10^3^ metacyclic parasites, 15 weeks post infection. Except for the production of IL-1β, which was reduced in *Gsdmd*^*–/–*^, we did not detect significant differences that support a crucial role of cytokines in the increased susceptibility of GSDMD-deficient mice.

**Fig. 8:**
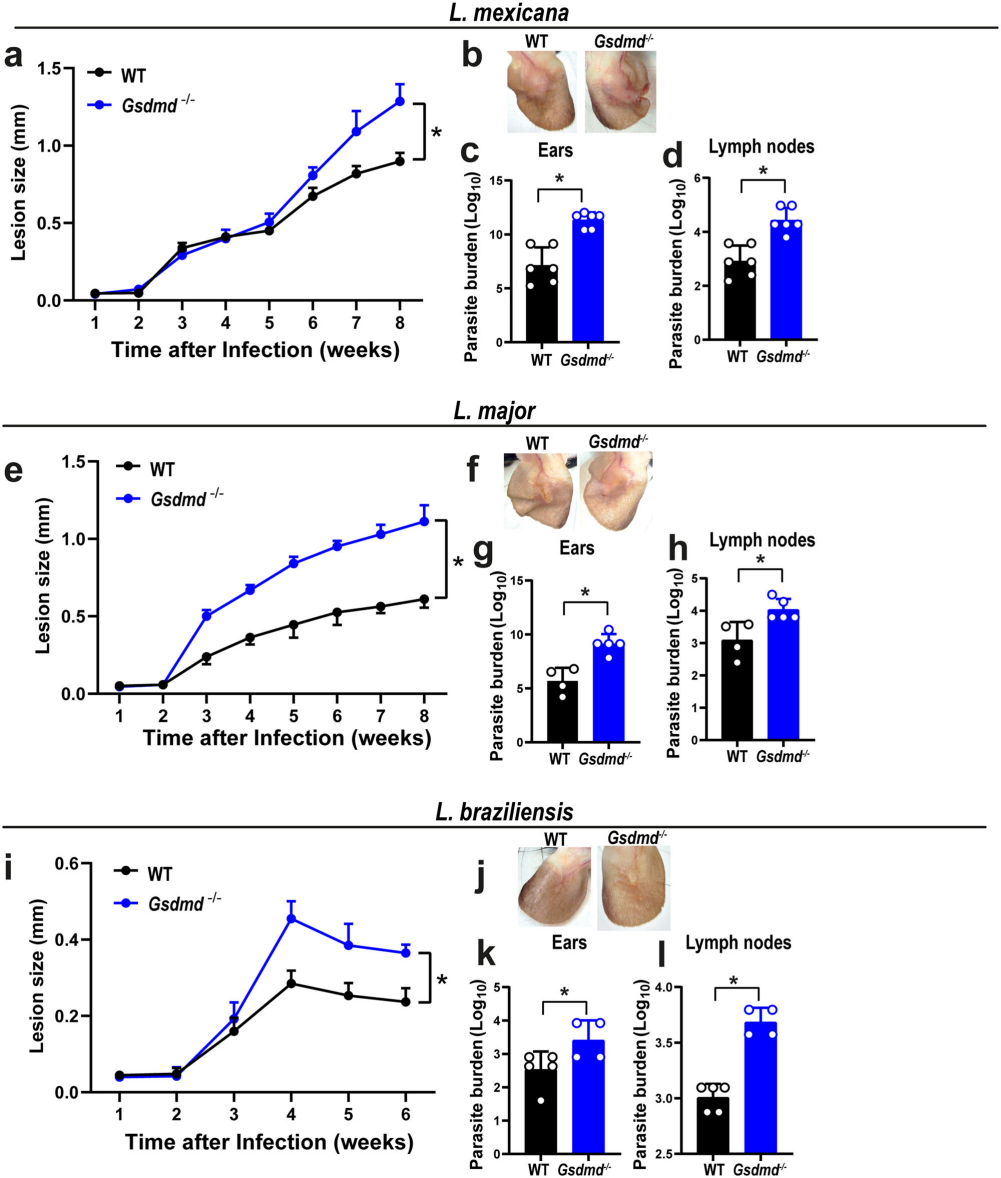
GSDMD is important for the restriction of *Leishmania* species in vivo*.* WT and *Gsdmd*^*–/–*^ mice were infected with 10^6^ stationary-phase *L. mexicana, L. major*, and *L. braziliensis* promastigotes in the ear, and the ear thicknesses were followed for 8 (**a**–**h**) or 6 weeks (**i**–**l**). **a**, **e**, **i** Lesion development; **b**, **f**, **j** images of infected ears; **c**, **d**, **g**, **h**, **k**, **l** limiting dilution assay to quantify parasite burden in the infected ears (**c**, **g**, **k**) and draining lymph nodes (**d**, **h**, **l**) at 8 or 6 weeks of infection. Each dot in the bar graphics represents the value obtained from an individual mouse. Data are presented as mean values ± SD. *P < 0.05 comparing the indicated groups, as determined by the Student *T* test two-sided. Shown is one representative experiment of two independent experiments performed. Source data are provided as a Source Data file.

Next, we assessed the role of GSDMD in host resistance to other species of *Leishmania* in vivo. In these experiments, mice were intradermally infected in the ear with 10^6^ stationary-phase promastigotes of *L. mexicana, L. major*, and *L. braziliensis*, and the lesions were measured up to 8 weeks after infection (6 weeks were used for *L. braziliensis* because mice in C57BL/6 genetic background are very resistant to this species). Our results show a significant increase in the lesion size and parasite burden in the ears and draining lymph nodes in *Gsdmd*^*–/–*^ mice compared to WT (Fig. 8), indicating an important role of GSDMD for host response against different species of *Leishmania*.

### NLRP3 inflammasome and GSDMD are active in infected humans

Next, we assessed the activation of GSDMD in the skin of patients with cutaneous Leishmaniasis. First, we evaluated gene expression in skin biopsies of seven patients infected with *Leishmania braziliensis* and compared them with five patients undergoing reductive mastectomy (control group). Demographic characterization of the patients is contained in Supplementary Table 1. No sex- and gender-based analysis was performed due to the low sample size. Expression of inflammasome and inflammatory genes in the patient’s skin was heterogeneous (Supplementary Fig. 6a). Still, our data demonstrated an overall increased expression of inflammasome-related genes such as *Gsdmd, Nlrp3, Casp1, Casp4, Il1b, Ifng, Tnfa, Il10*, and *Il17a* (Supplementary Fig. 6b–j) when we compared Leishmaniasis patients with controls. We did not detect statistically significant differences in the expression of *Il1ra, Il1a, Il18*, *Ifna1*, *Ifnb1*, *Aim2*, *Pycard*, *Il4*, *Il6*, *Nlrp1*, *Nlrc4* (Supplementary Fig. 6k–u). A correlation matrix indicated that the expression of GSDMD positively correlates with inflammasome and inflammatory genes, including *Il1b, Tnfa, Nlrp3, Il1a, Pycard, Nlrp1, Casp1*, and *Aim2* (Supplementary Fig. 6v).

**Fig. 9:**
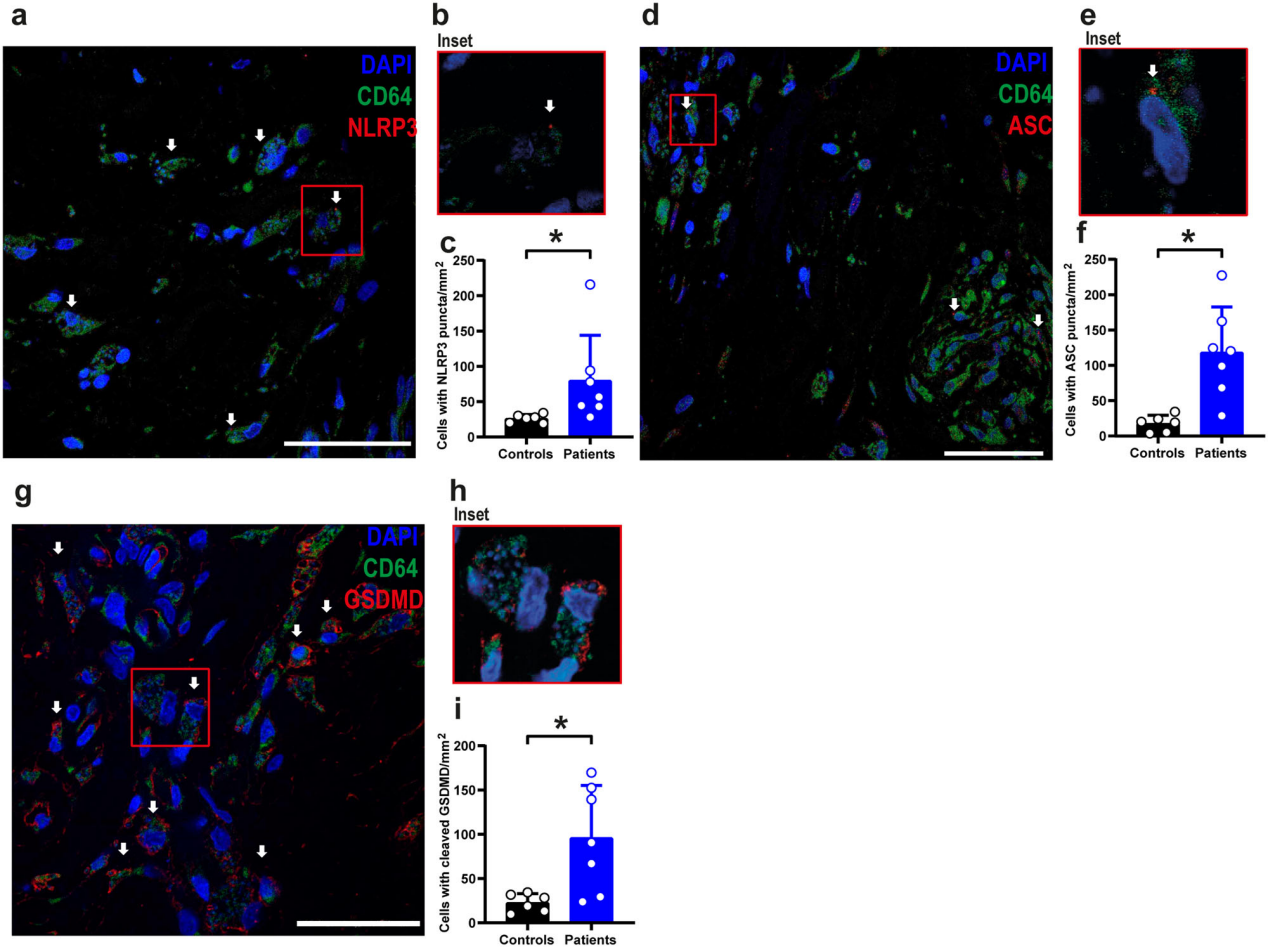
NLRP3/ASC inflammasome and GSDMD are active in skin biopsies of cutaneous leishmaniasis patients. Multiphoton microscopy of skin biopsies of seven Leishmaniasis patients and six healthy controls (skin from individuals who underwent reductive mastectomy). Tissues were stained with anti-NLRP3 (**a**–**c**), anti-ASC (**d**–**f**), and anti-cleaved GSDMD (N-terminal fragment) (**g**–**i**). Representative images showing NLRP3 puncta (**a**), ASC puncta (**d**), or cleaved GSDMD (**g**) in red, indicated by white arrows. DAPI stains cell nuclei (blue), and CD64 + cells are stained in green. Scale bar 40 µm. **b**, **e**, **h** Insets indicate a higher magnification of a region indicated (red rectangle). **c**, **f**, **i** Quantification of cells with NLRP3 (**c**) or ASC (**f**) puncta and cells with cleaved GSDMD (**i**) in skin biopsies. Each dot in the figure represents the value obtained from one individual. Data are presented as mean values ± SD. *P < 0.05 comparing the indicated groups, as determined by the Student *T* test two-sided. Source data are provided as a Source Data file.

To assess inflammasome activation in skin biopsies of Leishmaniasis patients, we stained the patient tissues with anti-ASC, anti-NLRP3, and cleaved GSDMD (N-terminal). We detected a robust inflammasome activation demonstrated by the formation of NLRP3 puncta (Fig. 9a–c), ASC puncta (Fig. 9d–f), and N-terminal GSDMD (Fig. 9g–i)﻿ in CD64^+^ cells. This data unequivocally demonstrates that inflammasome and GSDMD are activated in the skin lesions of cutaneous Leishmaniasis patients, supporting our data indicating the critical role of GSDMD for the outcome of Leishmaniasis.

## Discussion

**Fig. 10:**
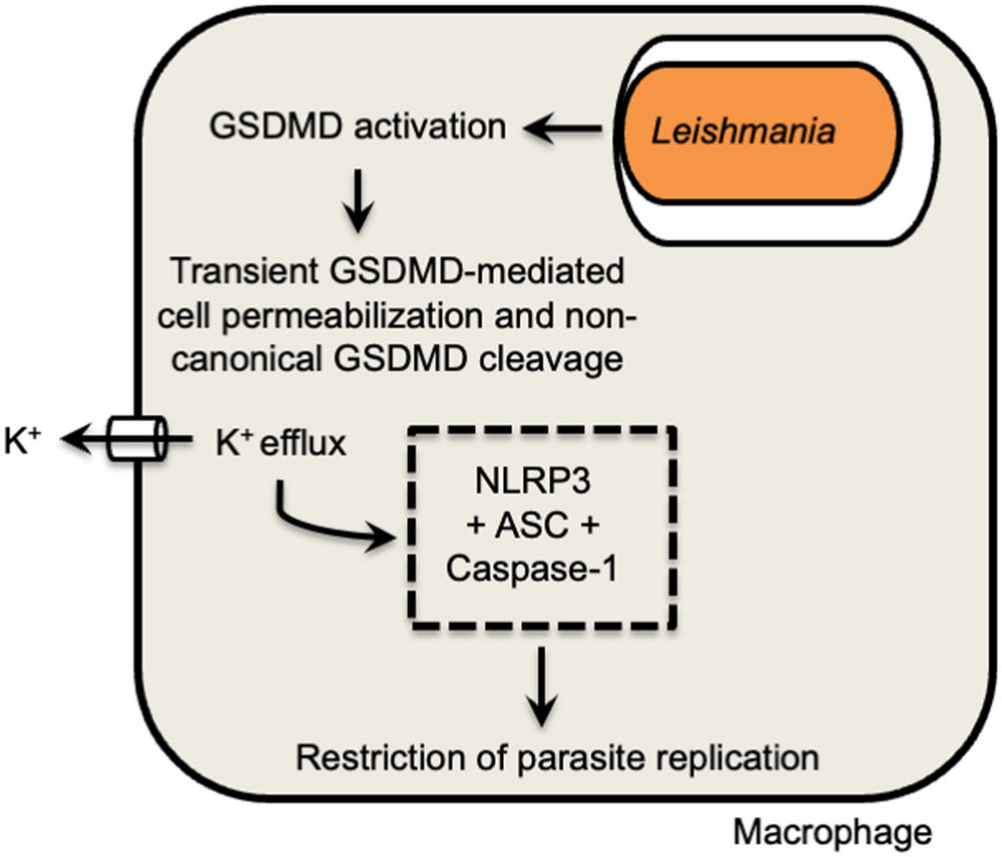
Schematic model illustrating the role of Gasdermin-D (GSDMD) in inflammasome activation and restriction of *Leishmania* replication in macrophages.

In this study, we reveal that GSDMD is activated in BMDMs and in lesions from patients with Leishmaniasis. This protein is important in restricting infection in macrophages and in vivo. Interestingly, our data show that upon *Leishmania* infection in BMDMs, there is a transient and weak activation of GSDMD, which may not be robust enough to promote cell death, but is sufficient to allow potassium efflux, enabling the noncanonical activation of NLRP3 (Fig. 10). This data effectively account to our understanding on how the inflammasome is activated in response to *Leishmania*, a process that is key to the outcome of leishmaniasis, but mechanistically it is still unclear. Importantly, this transient activation of GSDMD does not lead to cell death. This process may be actively manipulated by the parasites, which allows low levels of inflammasome activation without killing the macrophages, the main cells that harbor the replicating parasites. The low levels of inflammasome activation may guarantee continuous inflammation in infected skin, a process that may be sufficient to maintain inflammatory cells in the infection sites, accounting for the chronic and inflammatory nature of the lesions that are the hallmark of localized cutaneous Leishmaniasis.

Many groups have reported activating the inflammasome in response to *Leishmania* infection^12–14,16,17^. In response to other pathogens, this process usually leads to an inflammatory cell death, known as pyroptosis, which occurs via GSDMD^21,23^. Here, we show that GSDMD is activated in the first hours of *Leishmania* infection in BMDMs, although there is no significant difference in cell death rates 24 h post infection. Very intriguingly, we did not detect the canonical 30-kDa fragment of GSDMD characteristic of the cleavage by Caspase-1/4/11^21–23^, or by neutrophil elastase^41–43^. Instead, we detect the formation of a 25-kDa fragment, which is independent of caspases 1/7/8/11. The size of this cleaved fragment is also not consistent with GSDMD fragments cleaved by Caspase-8^44,45^, Caspase-3/7^46,47^, and the recently described 40-kDa fragment generated by allergen proteases^48^. It is possible that this alternative cleavage of GSDMD induced by *Leishmania* results in the inactivation of GSDMD-mediated pores, accounting to explain the transient GSDMD activation that occurred in the absence of cell death in the infected macrophages.

We also investigate the effect of ESCRT on cell death inhibition in *Leishmania*-infected cells. Cell transport machinery like ESCRT can repair the plasma membrane in damaging situations^32^ and this repair also occurs when pores are formed by GSDMD^33^, which inhibits GSDMD-mediated cell death. Our results suggest that the cell death inhibition seen in our study is not associated with a membrane repair system mediated by the ESCRT machinery (Supplementary Fig. 1). Therefore, another mechanism not yet identified should be involved in preventing cell death in *Leishmania* infection. Future studies may address the possible *Leishmania* interference in the Ragulator–Rag complex, a pathway described as necessary for induction of GSDMD pore formation^49^. Regardless of these yet unknown inhibitory mechanisms, it is expected that *Leishmania* evolved mechanisms to inhibit cell death as a mechanism for evading the immune response^7,8,10^. We show that GSDMD promotes transient membrane permeabilization in *L. amazonensis*-infected cells without causing cell death. It is still unknown whether a pore mediates it and how this permeabilization fails to promote cell death. *Leishmania has* several enzymes similar to caspases called metacaspases, although they have a catalytic site different from traditional caspases. The LmjMCA metacaspase is an example of caspase in *L. major* that has an important role in parasite apoptosis^50,51^. From the evolutionary perspective, there should be no selective pressure for the development of programmed cell death in unicellular organisms, such as *Leishmania*. Proteases such as LmjMCA may have a role in parasite development or subversion of host cell functions. Interestingly, LmjMCA has a mitochondrial localization sequence; its N- and C-terminal cleavages are important for the maturation of the catalytic domain, showing that this protein has enzymatic activity both in the cytoplasm and in mitochondria^51^. This dual repartition is similar for mammalian caspases. For example, caspase-3 is found in the cytosol and inner mitochondrial membrane, which is related to apoptotic signaling^52,53^. Thus, *Leishmania* metacaspase and/or other proteins with similar activity may be involved in the modulation of GSDMD functions in infected cells.

Despite the known importance of the inflammasome in infection control, there are still unanswered questions about the mechanisms involved in NLRP3 activation in *Leishmania-*infected macrophages. The production of ROS via Dectin-1 and potassium efflux is important for NLRP3 inflammasome activation^17,18,54^. In addition to these mechanisms, Carvalho et al. (2019) demonstrated that the noncanonical pathway of inflammasome has an important role in the activation of NLRP3 via LPG recognition^14^. Although no cell death is detected during *Leishmania* infection, we demonstrate that ASC puncta formation, PI entry, potassium efflux, IL-1β synthesis, and secretion are lower in *Gsdmd*^*–/–*^ BMDMs suggesting that GSDMD may contribute to the activation of NLRP3 inflammasome. This role of GSDMD in the noncanonical activation of the inflammasome has already been described in Gram-negative bacteria^21^. Further studies should investigate how this differential GSDMD cleavage takes place.

It has already been described that NLRP3 inflammasome is important for controlling *Leishmania* infection and that this process occurs partially through Caspase-11^13,14,17^. However, the GSDMD role has not been evaluated in *Leishmania* infection in vivo. We show for the first time that the absence of GSDMD leads to increased susceptibility to infection by *Leishmania* in mice and macrophages. This data highlights the pivotal role of GSDMD in response to *Leishmania* infection and provide a mechanism to explain the *Leishmania*-induced noncanonical activation of the NLRP3. The most important form of leishmania for in vivo infection is the amastigote, which despite having little LPG expression, is also capable of inducing inflammasome activation in macrophages^14^, in a process partially dependent on GSDMD. In addition, other inflammasomes may be involved in this process (AIM2, NLRP1, etc.), which could activate caspase-1 and GSDMD independently of LPG. Importantly, GSDMD can also be activated independently on inflammasomes. It was demonstrated that Caspase-8 could activate GSDMD in response to bacteria^44,45^. In addition, GSDMD was reported to be cleaved by neutrophil elastase^42,43^, a process that is inflammasome-independent and contributes to the death of neutrophils, a cell type that is very important in Leishmaniasis^12,55–58^. In this scenario, the deletion of GSDMD also abrogates inflammasome-independent pathways that operate to restrict *Leishmania* infection. This may explain the exacerbated susceptibility of the *Gsdmd*^*–/–*^ mice to *L. major* LV39 strain, a strain previously shown to be restricted in C57BL/6 mice independently of inflammasome components^17^.

Regardless of the pathways involved in GSDMD activation, we show a very important role for GSDMD in activating the NLRP3 inflammasome and restriction of *Leishmania* infection. Importantly, our experiments with clinical samples demonstrated activation of the inflammasome and GSDMD in the lesions of Leishmaniasis patients, supporting our assertion of the importance of GSDMD and inflammasomes in the course of the disease. Despite demonstrating the critical role of GSDMD in the disease outcome, our results indicate the existence of a still unknown pathway used by *Leishmania* to bypass GSDMD-mediated macrophage death. This process may involve processing GSDMD into the 25 kDa fragment, which may explain the transient pore formation reported herein. In summary, our studies show GSMDM as a central molecule involved in *Leishmania* interaction with macrophages, a key process for disease development. Further investigation of this process may reveal relevant drug targets in the fight against Leishmaniasis.

## Methods

### Animals and infection

Female or male mice aged between six and eight weeks were used. Mice include C57BL/6 (WT—Jackson Laboratory, stock number 000664) and strains deficient in caspase-11 (*Casp11*^*–/–*^)^59^, caspase-1/11 (*Casp1/11*^*–/–*^)^60^, NLRP3 (*Nlrp3*^*–/–*^)^61^, and Gasdermin-D (*Gsdmd*^*–/–*^)^46^ in the C57BL/6 background were maintained under specific pathogen-free conditions at the Ribeirão Preto Medical School of the University of São Paulo (FMRP/USP). Mice were maintained in ventilated cages in an ambient temperature and humidity-controlled room with a 12 h light/12 h dark cycle with continuous access to food and water. The experiments using animals were performed according to the rules of the Institutional Ethics Committee for Animal use, CETEA/FMRP (Comissão de Etica em Experimentação Animal da Faculdade de Medicina de Ribeirão Preto, Approved Protocol 018/2019).

### Parasite strains and cultivation

The parasites used in this study are *Leishmania (L.) amazonensis* PH 8 (IFLA/BR/67/PH 8) and this strain constitutively expressing RFP (kindly provided by Prof. Dr. David L. Sacks, National Institute of Allergy and Infectious Diseases (NIAID-NIH, USA), *Leishmania (V.) braziliensis* M2903 strain (MHOM/BR/75/M2903), *Leishmania (L.) major* LV39 strain (MRHO/SU/59/P), *Leishmania (L.) mexicana* M379 strain (MNYC/BZ/62/M379). *Leishmania braziliensis* and *Leishmania mexicana* strains constitutively expressing enhanced green fluorescent protein (EGFP) gene was constructed by amplifying EGFP from pEGFP-C1 using the primers NotI_FP_C1_F (GCGGCCGCATGGTGAGCAAGGGCGA) and BamHI_FP_C1_R (TGGATCCTTATCCCGGGCCCGCGGTACC). The EGFP was cloned into the plasmid pSSU-tdTomato-Neo^62^, originating pSSU-EGFP-C1-Neo. The pSSU-EGFP-C1-Neo was double digested with *Pac*I and *Pme*I, and the linear DNA fragment was used in the transfection process. After G418 positive selection, the transfectant clones were screened by PCR with the following primers: Neo_end_F (CTATCGCCTTCTTGACGAGTTCTTCTG) and Down_SSU_R (AGGAAGCCAAGTCATCCATC). The EGFP expression in promastigotes was confirmed by fluorescence microscopy. *Leishmania (L.) major* LV39 strain (MRHO/SU/59/P) constitutively expresses DSRed was kindly provided by Dr. Angela Cruz (FMRP/USP, Brazil).

These parasites were grown at 26 °C in Schneider medium (Invitrogen, Carlsbad, CA), pH 6.5, supplemented with 20% bovine fetal serum (SFB) (GIBCO BRL), 100 U ml − 1 penicillin G (USB Corporation, Cleveland, OH, USA), 2 mM l-glutamine and 1 mg/ml biopterin (Sigma). After six passages in culture, the parasites were passed on to a C57BL/6 mouse to maintain the strain’s virulence. The infectious forms of *L. amazonensis* metacyclic promastigotes were isolated from the stationary-phase cultures through serial centrifugations at 80×*g* and then at 2000×*g*. *Leishmania* in the metacyclic phase was isolated by density gradient using ficol 40% and 10% according to the protocol described^63^.

### Preparation of BMDMs and in vitro infection

Bone marrow-derived macrophages (BMDMs) were differentiated from cells extracted from mouse femurs C57BL/6, *Nlrp3*^*–/–*^, *Casp1*/*11*^*–/–*^, *Casp11*^*–/–*^, and *Gsdmd*^*–/–*^. Bone marrow cells were differentiated into R10/20 medium comprising RPMI 1640 containing 20% SBF, 30% conditioned medium from LCCM cells, 15 mM HEPES, 2 g NaHCO_3_, 100 U/ml penicillin, 100 μg/ml streptomycin and 2 mM l-glutamine. The cells were kept in Petri dishes of dimensions 90 ×15 mm (Becton Dive—BD, USA), at 37 °C, in a humidified atmosphere containing 5% CO_2_. After 4 days, an additional 10 ml of the respective medium was added. After seven days of incubation, macrophages were collected by washing the monolayers with ice-cold phosphate-buffered saline (PBS). The cells were placed in 6-, 12-, 24-, or 96-well plates in R10 medium (RPMI containing 10% SBF) and allowed to adhere for 24 h before being used in the experiments. After 24 h, BMDMs were infected with promastigote forms of the parasite in the stationary phase, with MOI 10. After 1, 2, or 4 h of infection (as indicated in the legend), the infected cultures were washed with PBS at 37 °C and 10% RPMI culture medium (10% serum fetal bovine).

### Retroviral transduction of GSDMD-mNeon

The lentiviral vector encoding GSDMD-mNeon-tagged^31^ was used to express GSDMD fused with mNeon in macrophages. Vectors psPAX2 and PMDG were used for retroviral packaging in HEK Peak cell monolayers (ATTC CRL-2828) transfected with the three plasmids. The Peak cell supernatant was collected 3 days after transfection, filtered through a 0.45-μm filter, and added to the BMDM cultures after 3 days of differentiation. BMDMs were obtained from the femur of *Gsdmd*^*–/–*^ mice cultured in RPMI 1640 medium supplemented with 20% SFB and 30% supernatant from LCCM cells. After 3 days of differentiation, BMDMs were collected and placed in a culture containing Peak cell supernatant and supplemented with RPMI containing 20% SFB with 30% LCCM. The BMDMs were again fed on the 5th day of culture, with RPMI 1640 medium containing 30% LCCM and 20% SFB and the cells expressing the plasmids were selected using 1 mg/ml of G418. After 7 days of culture, the monolayer of macrophages transduced for expression of GSDMD-mNeon was detached with cold PBS and placed in 24-well plates containing 13 mm coverslips. Thus, the culture of macrophages expressing GSDMD-mNeon was infected with *L. amazonensis*, constitutively expressing RFP at an MOI 5.

### Immunofluorescence

For in vitro experiments, BMDMs have been placed in 13-mm coverslips at the confluence of 2 × 10^5^ cells/well, in 24-well plates. The cells were infected with *L. amazonensis* expressing the red fluorescent protein (RFP). The plates were centrifuged at 200×*g* for 5 min at room temperature and incubated for 24 h at 37 °C and 5% CO_2_. The plasma membrane was stained with Alexa Fluor® 647 conjugate of Wheat Germ Agglutinin (WGA) (Invitrogen; W32466) following the manufacturing instructions. The cells were then fixed with 4% paraformaldehyde and permeabilized with 0.1% saponin. The coverslips were washed with PBS and blocked for 1 h, at room temperature, with PBS containing 10% goat serum. The ASC puncta were stained with rabbit anti-ASC antibody (1:2000; Adipogen AL177) or rabbit anti-cleaved C-terminal GSDMD antibody (1:1000; Abcam; ab255603), followed by staining with secondary antibody conjugated with Alexa-488 (dilution 1: 3000, Invitrogen, A-11008) or goat anti-rabbit 594 (dilution 1:3000, Invitrogen, A-11012). All washes of antibodies were done in PBS, and all dilutions were in PBS-Goat Serum. The coverslips were inverted in a mounting medium containing DAPI (DAPI Prolong® Gold antifade reagent, Invitrogen) on slides for fluorescence microscopy. The pore formation of GSDMD-mNeon and ASC puncta was evaluated by observing the number of 100 cells in 63x magnification under the Multifoton Zeiss LSM 780 microscope and Zen Black software (Carl Zeiss, Jena, Germany). The percentage of cells showing ASC Puncta was obtained, and the standard deviations were calculated from three coverslips.

For immunofluorescence of human skin biopsy and mouse-infected ears, tissue samples of 3-µm thickness were stained with hematoxylin and eosin (H&E) and analyzed by pathologists. Other sections of 3-µm thickness were incubated with the primary antibodies overnight at 4 °C and with the secondary antibodies Goat anti-mouse Alexa fluor-647 (dilution 1:200, Invitrogen, A-21235) or goat anti-rabbit Alexa fluor-594 (dilution 1:200, Invitrogen, A-11012). Images were acquired by the Axio Observer system combined with the LSM 780 confocal device microscope at ×63 magnification (Carl Zeiss) and Zen Black software (Carl Zeiss, Jena, Germany). For puncta counts, all histological sections were viewed on a ×63 objective for digitizing random images using the LSM 780 system in the Axio Observer microscope, was analyzed an area of ~1.7 mm^2^ per case. Manual counting of punctate and cells was performed using the acquired images. Morphometric analyses were performed as described^64,65^.

### ELISA assay

For the quantification of cytokines by ELISA (Enzyme-Linked ImmunoSorbent Assay), BMDMs were plated in 24-well plates (5 × 105 cells/well) and pretreated with 100 ng/ml LPS (Escherichia coli 055: B5 LPS, Sigma) or Pam3Cys for 4 h and then infected with *L. amazonensis* (MOI 10). The cytokine IL-1β present in the supernatant was measured by ELISA using the BD OptEIA kits (BD Biosciences), according to instructions provided by the manufacturer. The OD values were read on the SpectraMax i3 system (Molecular Devices).

### Membrane permeabilization assays

To assess the influx of propidium iodide (PI) by flow cytometry, BMDMs at 5 × 10^5^ cells/well were cultured in 24-well plates. The cells were infected with *L. amazonensis* with MOI 10 and centrifuged at 200 × *g* for 5 min, 25 °C. After 4 h and 24 h, the cells were treated with R10 medium containing propidium iodide (5 μg/ml) for 30 min, after which time the plates were washed with PBS and the cells were released with trypsin. The fluorescence of cells labeled with propidium iodide was detected in a flow cytometer in the FL3 channel. To assess the influx of propidium iodide (PI) by immunofluorescence, *Leishmania* was added at an MOI 10 to 5 × 10^5^ BMDMs plated on 13-mm glass coverslips in 24-well tissue culture dishes. Before the infection, the cells were treated with 100 ng/ml of LPS or Pam3Cys for 4 h. After the infection, the plates were centrifuged for 200×*g* for 5 min at room temperature and incubated for 2 h or 24 h at 37 °C in 5% CO2. The coverslip was then inverted onto a 5 µL drop of PBS containing 25 µg/ml PI and 5 µg/ml Hoechst. All cells were stained with Hoechst, whereas only cells with membrane pores allowed diffusion of PI into the cell. Images were acquired using Leica microscope (DMI4000B) with 100x objectives and analyzed using ImageJ software.

### Intracellular K^+^ determination

Intracellular potassium concentrations were determined by fluorescence emission from the Assante Potassium Green-2 probe (APG-2 AM, TEFlabs) as previously described^66^. BMDMs from C57BL/6 and *Gsdmd*^*–/–*^ mice were plated in 96-well plates (Corning 96 Well Flat Clear Bottom Black Polystyrene TC-Treated Microplates) and infected by *L. amazonensis* for 2 or 24 h. After infection, the supernatant was removed and cultures were added in RMPI 1640 medium without phenol red and without SFB, containing 5 μM APG-2 AM for 30 min. After incubation with the probe, the wells were washed 3 times with PBS at room temperature and added to the end of the RPMI 1640 washes without phenol red and without SFB. Fluorescence was read on the SpectraMax i3 system (Molecular device) fluorometer. The parameters of the analysis of the Relative Fluorescence Units (RFU), were standardized to read the emission in the wavelength determined by the manufacturer.

### Parasite quantification in cells and tissues

The leishmanicidal activity of macrophages in vitro was determined by flow cytometry using the FACS ACCURI C6 flow cytometer (BD Biosciences) or counted on slides stained with Giemsa in a light microscope with ×40 magnification, at times 1, 24, 48, and 72 h after infection. Cytometry data were analyzed using the FlowJo software (Tree Star), two parameters were considered: the percentage of infected cells and the mean of fluorescence, which reflects the mean of amastigotes per cell. In Giemsa staining analysis, the infection rate was determined by the ratio of infected and uninfected cells (100–200 BMDMs) and the number of intracellular amastigotes per infected BMDM. For in vivo infections, the mice were infected with 10^6^ parasites either in stationary phase or 10^3^ *Leishmania* in the metacyclic phase as previously described^17^. The ear injury was measured weekly with a digital caliper and was compared with the thickness of the uninfected right ear. Limiting dilutions were used to assess the parasite loads in the infected tissues as reported^17^, Briefly, at the indicated week of infection, the mice were sacrificed and the infected ear and the draining lymph node were removed, macerated and plated in a 96-well culture plate with Schneider medium in a 1:20 serial dilution for the ear and 1:5 for the lymph node. After 7 days of culture at 26 °C, the number of parasites in the respective organ was counted according to the growth observed in the serial dilution.

### Parasite quantification and mRNA expression in infected mice

The mice were infected with 10^3^ *Leishmania amazonensis* in the metacyclic phase as previously described^17^, after 15 weeks the ear and lymph nodes were collected. DNA and RNA were obtained by extraction with 1 mL of Trizol reagent and purification were performed according to the manufacturer’s instructions. The RNA and DNA was quantified by spectrophotometry in a NanoDrop 2000c spectrophotometer. The concentration was adjusted to 1 µg/µL. The RNA was stored at −70 °C until reverse transcription and used for cytokines mRNA expression. DNA was used for parasite quantification by real-time PCR, using primers specific for parasite kDNA gene as previously described^67^. For reverse transcription, the total RNA was transcribed into complementary DNA (cDNA) using a High-Capacity cDNA Reverse Transcription kit (without an inhibitor) according to the protocol provided by the manufacturer (Thermo Fisher, Carlsbad, CA, USA). The reaction was prepared in a final volume of 20.0 µL containing 4.2 µL of H_2_O, 2.0 µL of buffer, 2.0 µL of random primers, 0.8 µL of dNTP Mix (100 mM), 1.0 µL of reverse transcriptase (RT) enzyme and 1 µL of RNA (1 ug/µL). The solution was then placed into a thermocycler with the following program: 25 °C for 10 min, 37 °C for 120 min and 85 °C for 5 min. The real-time PCR was performed in 96-well plates using Sybr Green reagents (Applied Biosystems, Waltham, MA, USA) and a Quant studio real-time PCR system (Applied Biosystems, Foster City, CA, USA). The real-time-RT-PCR was carried out in a total volume of 20 μl on a 96-well MicroAmp Fast Optical plate (Applied Biosystems). Each well contained 10 μl SYBR Green qPCR Master Mix (Thermofisher), 1 μl of each primer, 2 μl cDNA (20 ng) and 7 μl RNase-free water using the following protocol: initial denaturation at 95 °C for 10 min, 40 cycles of denaturation at 95 °C for 15 s followed by annealing/extension at 60 °C for 60 s. Primer sequences are in Supplementary Table 2. Each PCR was followed by a dissociation curve analysis between 60 and 95 °C. The Ct values were analyzed by the comparative Ct (ΔΔCt) method and normalized to the endogenous control GAPDH. Fold difference was calculated as 2^−ΔΔCt^.

### Ex vivo cytokine quantification

The mice were infected with 10^3^ *Leishmania amazonensis* in the metacyclic phase as previously described^17^, after 15 weeks the ears were collected and macerated in PBS. IL-1β levels were evaluated by ELISA assay (R&D Systems) in the ear macerate of infected mice following the manufacturer’s instructions. TNF-α, IL-4, IL-6, IL-10, IFN-γ, and IL-17 were quantified in the ear macerate using a mice cytometric bead array (CBA) cytokine kit (Th1/Th2/Th17 Cytokine Kit; BD Biosciences) following the manufacturer’s instructions.

### Western blot

A total of 10^7^ BMDMs were used per well, primed with 100 ng/ml of ultrapure LPS (InvivoGen, tlrl-peklps) for 4 h and then infected with *L. amazonensis* for 24 h. The supernatants were collected and concentrated by ultrafiltration (Amicon Ultra 0.5-mL centrifugal filters). After clarification by centrifugation, cells was lysed with RIPA buffer (10 mM Tris-HCl, pH 7.4, 1 mM EDTA, 150 mM NaCl, 1% Nonidet P-40, 1% deoxycholate and 0.1% SDS) in the presence of protease inhibitor cocktail (Roche). The lysate and supernatant were solubilized in a heated Laemmli buffer, added to the SDS-PAGE and transferred (Semidry Transfer Cell, Bio-Rad) to a 0.22-μm nitrocellulose membrane (GE Healthcare). The membrane was blocked with Tris-buffered saline (TBS) with 0.01% Tween-20 and 5% skimmed milk powder. Monoclonal antibodies to IL-1β (dilution 1: 1000, Sigma, I3767), ASC (dilution 1:1000, Adipogen AL177) and GSDMD (dilution 1: 1000, Abcam, Ab209845) and specific secondary antibodies IRDye 800CW Donkey anti-Goat IgG (H + L) (LI-COR, 926-32214, dilution 1:10,000), IRDye 680RD Donkey anti-Mouse IgG (H + L) (LI-COR, 926-68072, dilution 1:10,000), goat anti-mouse HRP (KPL, 074-1806, dilution 1:3000), goat anti-rabbit HRP (Sigma, A6154, dilution 1: 3000) and rabbit anti-goat HRP (KPL, 14-13-06, dilution 1:3000) were diluted in blocking buffer for incubations. Detection of infrared secondary antibodies was visualized by the LI-COR Odyssey CLx imaging system. The ECL luminol reagent (GE Healthcare) was used for the conventional chemiluminescence antibody detection. All the blots shown are full scan blots.

### LDH release assay

Supernatant from 5 × 10^5^ BMDMs was collected, and the activity of released LDH was measured using colorimetric assays with CytoTox 96® (Promega) according to the manufacturer’s instructions. The percentage of LDH release was calculated as the ratio of the OD 490-nm sample/maximum OD. A maximum was obtained from BMDMs lysed with Triton X100. The OD values were read on SpectraMax i3 system (Molecular Devices).

### Video acquisition

To assess the influx of propidium iodide (PI) by time-lapse microscopy, BMDMs at 25 × 10^4^ cells/well were cultured in 30-mm plates. Before the infection, the cells were treated with 100 ng/ml de LPS or Pam3Cys for 4 h. The cells were infected with *L. amazonensis* with MOI 10 and centrifuged at 200×*g* for 5 min, 25 °C with R10 medium containing propidium iodide (5 μg/ml), after centrifugation the plates are incubated in the BioStation IM-Q microscopy (Nikon) and images were acquired every 10 min for 20 h at phase contrast and 594 channel. Images were analyzed using ImageJ software.

### ASC oligomerization assay

A total of 10^7^ BMDMs were used per well, primed with 100 ng/ml of ultrapure LPS (InvivoGen, tlrl-peklps) for 4 h and then infected with *L. amazonensis* for 24 h. BMDMs were resuspended in a hypotonic solution (10 mM Hepes – pH 7.9, 1.5 mM MgCl_2_, 10 mM KCl, 0.2 mM PMSF, 0.5 mM DTT, protease inhibitor cocktail Roche), incubated on ice for 15 min, homogenized (Kontes 22 mm) and centrifuged for 8 min at 10,000×*g*. The pellets were resuspended in 500 µl of CHAPs buffer (20 mM HEPES-KOH – pH 7.5, 5 mM MgCl_2_, 0.5 mM EGTA, 0.1% CHAPs, 0.1 mM PMSF, and protease inhibitor cocktail from Roche) and centrifuged for 8 min at 10,000×*g*. Finally, the pellets were resuspended in 200 µl of CHAPs buffer, 4 µl of a 100 mM DSS stock solution to a final concentration of 2 mM, and incubated for 30 min in the dark. The oligomers were resolved on a 12% SDS-PAGE and visualized by immunoblotting with an anti-ASC antibody (Adipogen AL177, 1:1000).

### Skin biopsy samples

Samples from patients infected with *L. braziliensis*, were obtained from the Serviço de Patologia (SERPAT) of the Hospital das Clínicas da Faculdade de Medicina de Ribeirão Preto da Universidade de São Paulo, Brazil, seven tissue samples embedded in paraffin and fixed in formalin (formalin-fixed paraffin-embedded, FFPE) of skin biopsies from the lesion of patients diagnosed with cutaneous leishmaniasis, which were collected between 2010 and 2019. The control group consisted of six tissue samples embedded in paraffin and fixed in formalin (formalin-fixed paraffin-embedded, FFPE) from biopsies of skin of patients who have undergone reductive mastectomy. The quantifications performed using human samples were approved by the Human Research Ethics Committee of USP-HCFMRP CEP (Universidade de São Paulo, Hospital das Clínicas da Faculdade de Medicina de Ribeirão Preto, Comissão de Ética em Pesquisa, under protocol number 5.117.488). As human samples were already available in the SERPAT-HC/FMRP, written informed consent was not necessary for approval of this study by the Human Research Ethics Committee of USP-HCFMRP.

### RNA extraction, reverse transcription, and real-time PCR

RNA was extracted from paraffin-embedded tissue samples. Samples were cut into three sections of 10 μm, and placed in RNase-free, 2.0-ml Eppendorf tubes. Sections were deparaffinized by incubation in 0.8 ml of xylene at 37 °C for 5 min. The samples were then centrifuged, the supernatant was removed, and fresh xylene was added for a second incubation. After deparaffinization and centrifugation, sections were washed with 0.8 ml ethanol, air-dried for several minutes, and resuspended in 25 μl of 20 mg/ml Proteinase K (Gibco BRL, Gaithersburg, MD) plus 720 μl of a digestion buffer with the following final concentrations: 20 mM TRIS-HCl ph 8, 10 mM EDTA pH 8, 1% of SDS. Samples were vortexed and incubated overnight at 55 °C at 20 g. A second 25 μl aliquot of 20 mg/ml Proteinase K was then added, followed by vortexing and incubation for 2 h at 55 °C. RNA was obtained by extraction with 1 mL of Trizol reagent and purification was performed according to the manufacturer’s instructions. The RNA was quantified by spectrophotometry in a NanoDrop 2000c spectrophotometer. The concentration was adjusted to 1 µg/µL, and the RNA was stored at −70 °C until reverse transcription. For reverse transcription, the total RNA was transcribed into complementary DNA (cDNA) using a High-Capacity cDNA Reverse Transcription kit (without an inhibitor) according to the protocol provided by the manufacturer (Thermo Fisher, Carlsbad, CA, USA). The reaction was prepared in a final volume of 20 µL containing 4.2 µL of H2O, 2.0 µL of buffer, 2.0 µL of random primers, 0.8 µL of dNTP Mix (100 mM), 1.0 µL of reverse transcriptase (RT) enzyme and 1 µL of RNA (1 ug/µL). The solution was then placed into a thermocycler with the following program: 25 °C for 10 min, 37 °C for 120 min, and 85 °C for 5 min. The real-time PCR was performed in 96-well plates using Sybr Green^TM^ reagents (Applied Biosystems, Waltham, MA, USA) and a Quant studio real-time PCR system (Applied Biosystems, Foster City, CA, USA). The real-time-RT-PCR was carried out in a total volume of 20 μl on a 96-well MicroAmp Fast Optical plate (Applied Biosystems). Each well contained 10 μl SYBR Green qPCR Master Mix (Thermofisher), 1 μl of each primer, 2 μl cDNA (20 ng), and 7 μl RNase-free water using the following protocol: initial denaturation at 95 °C for 10 min, 40 cycles of denaturation at 95 °C for 15 s followed by annealing/extension at 60 °C for 60 s. Each PCR was followed by a dissociation curve analysis between 60 and 95 °C. The Ct values were analyzed by the comparative Ct (ΔΔCt) method and normalized to the endogenous control GAPDH. The fold difference was calculated as 2^−ΔΔCt^.

### Statistical analysis

For the comparisons of multiple groups, two-way analysis of variance (ANOVA), followed by the Bonferroni post-test were used. The differences in the values obtained for two different groups were determined using an unpaired, two-tailed Student’s *t* test with 95% confidence interval. Analyses were performed using the GraphPad PRISM 5.0 software (GraphPad, San Diego, CA).

Correlations between the fold change differences for the cytokines analyzed were assessed by Spearman correlation and are represented in the correlation matrix. Only values with *P* < 0.05 are indicated in the matrix. The matrix correlation was constructed using the GraphPad PRISM 5.0 software (GraphPad, San Diego, CA) and represents the correlation scores, which were categorized as Strong Positive Correlation (*r* ≥ 0.7), Moderate Positive Correlation (0.5 ≥ *r* ≤ 0.7), Weak Positive Correction (0.3 ≥ *r* ≤ 0.5), No Correlation (–0.3 ≥ *r* ≤ 0.3), Weak Negative Correlation (–0.5 ≥ *r* ≤ -0.3), Moderate Negative Correlation (–0.7 ≥ *r* ≤ –0.5), Strong Negative Correlation (*r* ≤ –0.7).

We generated hierarchical clustering of patients based on all values of fold change difference, calculated as 2 − ΔΔCt. Hierarchical clustering was based on a correlation distance measure using ward.D2 and Canberra method. Heatmaps were constructed using the heatmap.2 function in the R studio (Version 1.3.1073; R studio, Boston, MA).

### Reporting summary

Further information on research design is available in the Nature Portfolio Reporting Summary linked to this article.

## Supplementary information

Supplementary information

Description of additional supplementary files

Supplementary Movie 1

Supplementary Movie 2

Supplementary Movie 3

Reporting Summary

## Data Availability

The data generated in this study are provided in the Supplementary Information/Source Data file. Plasmid and generated *Leishmania* strains are available via MTA from the corresponding author. Source data are provided with this paper.

## Source data

Source data
