# Supplementary material for: Gasdermin-D activation promotes NLRP3 activation and host resistance to Leishmania infection
Source: Nat Commun. 2023 Feb 24;14:1049. doi: 10.1038/s41467-023-36626-6 (PMC9958042; doi:10.1038/s41467-023-36626-6)
Supplement: Supplementary file 1 — Supplementary information [file 41467_2023_36626_MOESM1_ESM.pdf]

Gasdermin-D activation promotes NLRP3 activation and host resistance to *Leishmania* infection

Keyla S.G. de Sá et al.

Supplementary Figures 1-6 (pages 2-8):

Supplementary Table 1 (page 9):

Supplementary Table 2 (pages 10 and 11):

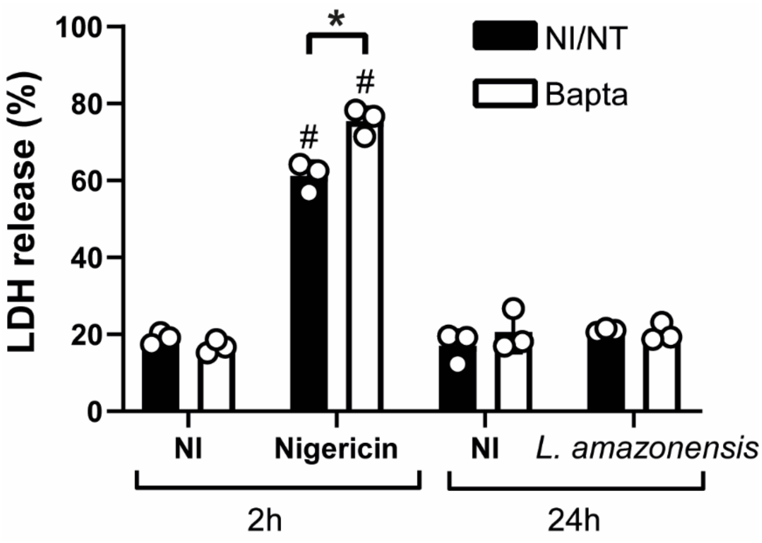

Supplementary Fig. 1. BAPTA does not interfere with LDH release in *Leishmania*-infected macrophages. Bone marrow-derived macrophages (BMDMs) from C57BL/6 mice were pretreated for 4h with LPS (100 ng/mL) and BAPTA (3µM) and infected with *L. amazonensis* at an MOI 10 or treated with Nigericin. LDH release was assessed 24h after infection with *Leishmania* and 2h after treatment with Nigericin using CytoTox 96 NonRadioactive Cytotoxicity Assay. Data are presented as mean values ± SD of triplicate wells. #, $P < 0.05$ compared with NI or NT cells; *, $P < 0.05$ comparing the indicated groups, as determined by the Two-way ANOVA test. Shown is one representative experiment of three independent experiments performed.

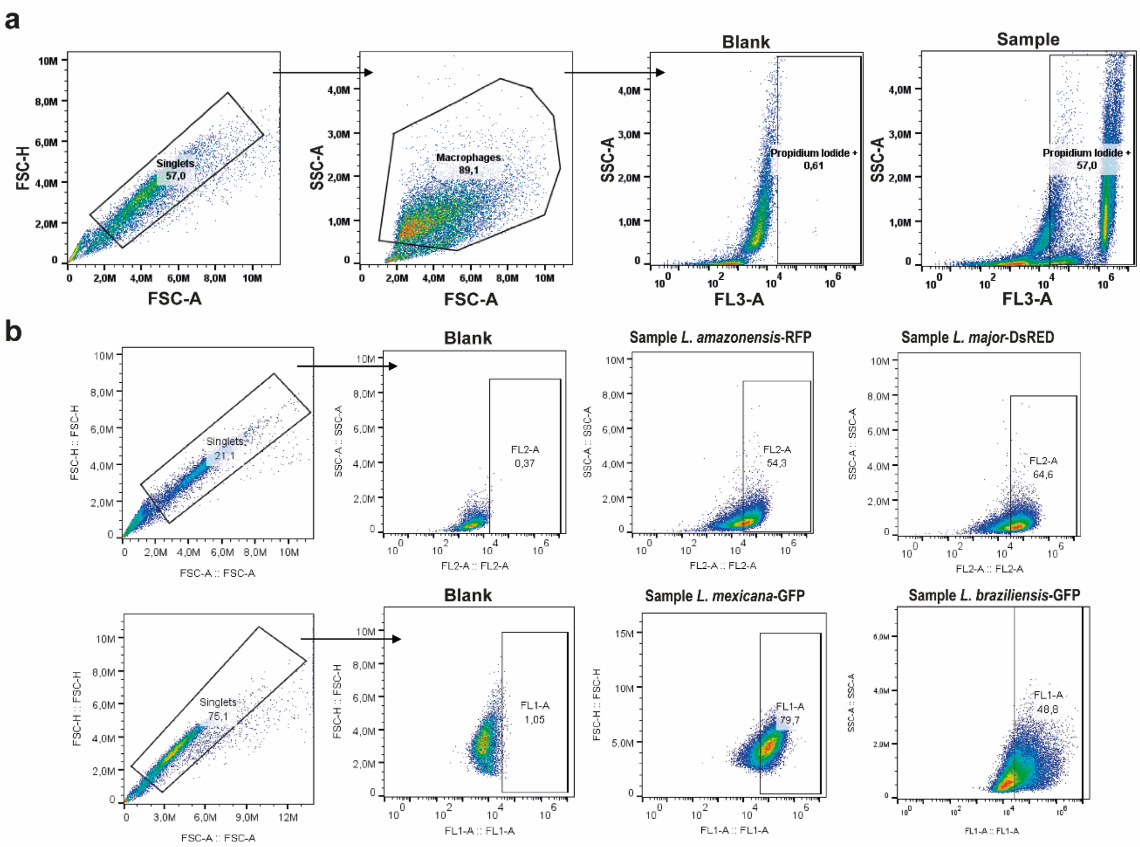

Supplementary Fig. 2. Gating strategy for flow cytometry. (a) Gating strategy for flow cytometer to analyze the PI uptake in *Leishmania*-infected cells. **(b)** Gating strategy for flow cytometer to analyze the *L. amazonensis*-RFP, *L. major DsRED*, *L. mexicana-GFP* and *L. braziliensis*-GFP replication.

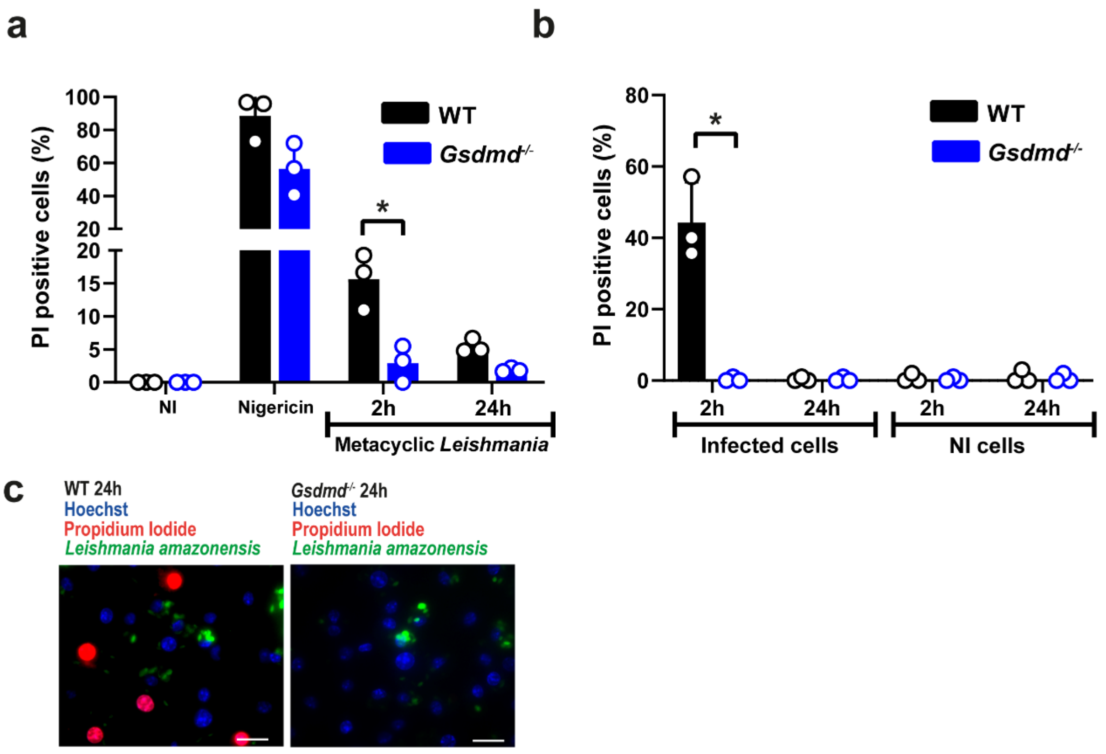

Supplementary Fig. 3. Metacyclic promastigotes and axenic amastigotes of *L. amazonensis* induce GSDMD-dependent membrane permeabilization in macrophages. Bone marrow-derived macrophages (BMDMs) adherent to glass coverslips was pretreated for 4h with Pam3Cys (100 ng/mL) and infected with GFP-expressing metacyclic *L. amazonensis* at an MOI 1 for 2h or 24h; the cultures were processed for pore formation assay by staining with propidium iodide (PI) and Hoechst. (**a**) The percentage BMDMs with PI incorporation after 2 and 24hs infection with *L. amazonensis*. (**b**) The percentage BMDMs with PI incorporation in infected and non-infected cells. A total of 100 cells in each triplicate well were analyzed. (**c**) Representative images of PI^+ cells (red), Hoechst (blue), and *Leishmania* (green). Images were acquired by fluorescence microscopy with a 100x oil immersion objective and analyzed using ImageJ software. Scale bar 20µm. Data are presented as mean values ± SD of triplicate wells. #, $P < 0.05$ compared with NI cells, as determined by Two-way ANOVA.; *, $P < 0.05$ comparing the indicated groups, as determined by Two-way ANOVA. Shown is one representative experiment of five independent experiments performed.

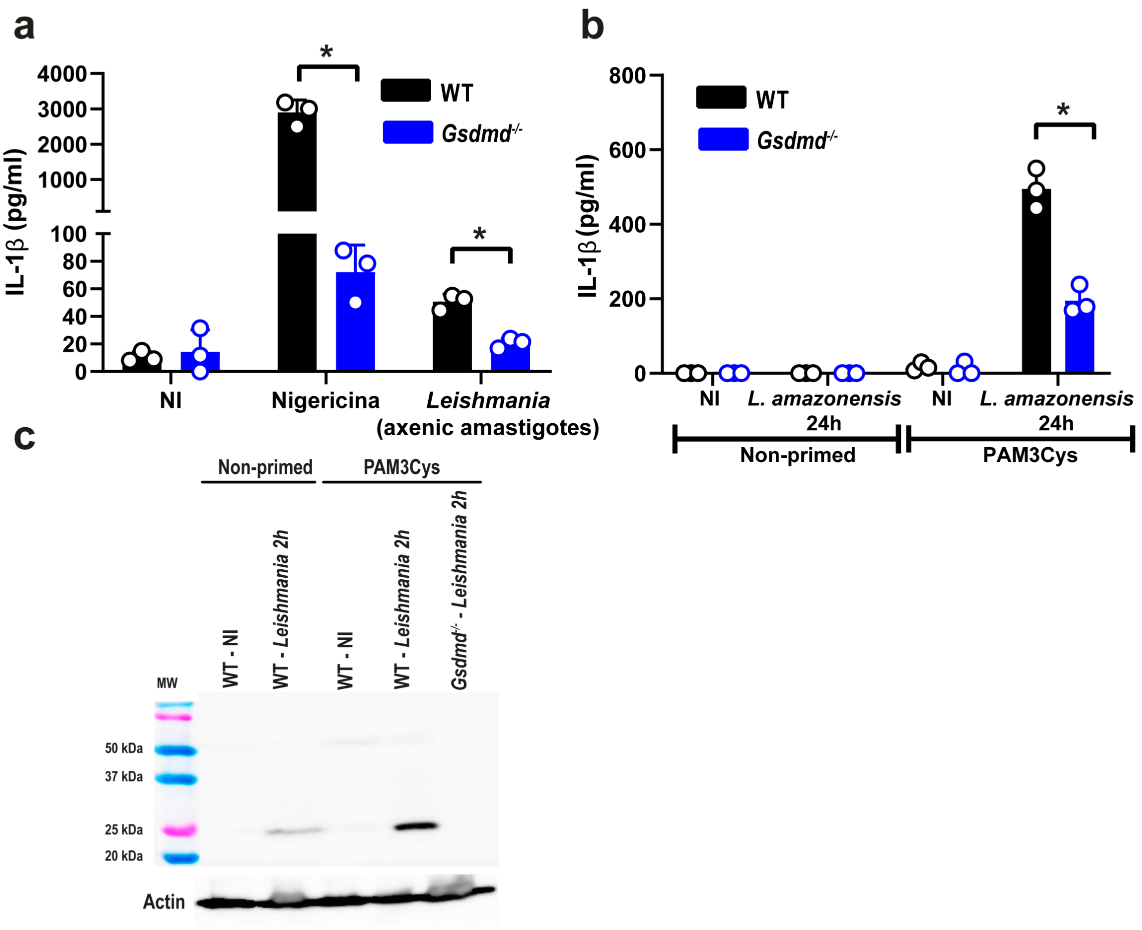

Supplementary Fig. 4. Amastigotes and metacyclic forms of *L. amazonensis* induce GSDMD-dependent inflammasome activation when primed with Pam3Cys. (a-b) Bone marrow-derived macrophages (BMDMs) from C57BL/6 (WT) and *Gsdmd*$^{-/-}$ mice were treated or not with Pam3Cys (100 ng/mL) for 4h, and infected with axenic amastigotes forms of *L. amazonensis* with an MOI 1 (**a**) or stationary phase promastigotes of *L. amazonensis* at an MOI of 10 (**b**) for 24 h. IL-1β production was measured by ELISA. Data are presented as mean values ± SD of triplicate wells. (**c**) Lysates from WT and *Gsdmd*$^{-/-}$ BMDMs pretreated or not with Pam3Cys (100 ng/mL) for 4h were left non-infected (NI) or infected for 2h with *L. amazonensis* (stationary phase promastigotes, MOI 10). GSDMD cleavage was assessed by western blot using an anti-GSDMD antibody. Full-length (55 kDa) and cleaved (25 kDa) GSDMD are indicated in the figure. MW, Molecular Weight.

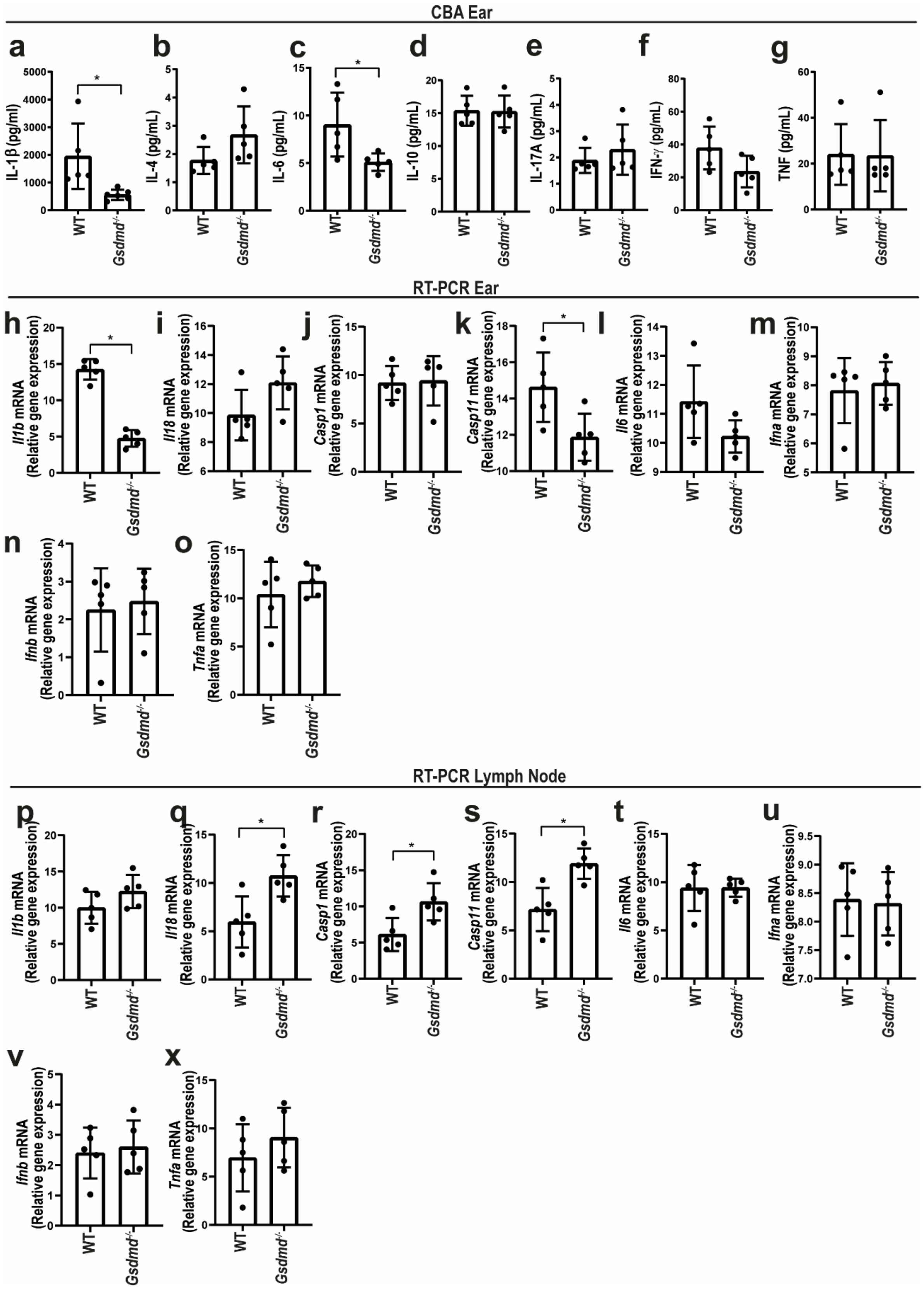

Supplementary Fig. 5. Inflammatory cytokines expression in the ear and lymph nodes of WT and *Gsdmd*$^{-/-}$ mice infected with *L. amazonensis*. (**a-g**) Levels of

cytokines (quantified by CBA) in the ears of mice intradermally infected with 10^3 metacyclic *L. amazonensis* for 15 weeks. Selected cytokines were IL-1β (**a**), IL-4 (**b**), IL-6 (**c**), IL-10 (**d**), IL-17A (**e**), IFNγ (**f**) and TNFα (**g**). Expression of cytokines mRNA in the ears and lymph nodes of WT and *Gsdmd*$^{-/-}$ mice infected with 10^3 metacyclic *L. amazonensis* for 15 weeks. Selected genes were *Il1b* (**h, p**), *Il18* (**i, q**), *Casp1* (**j, r**), *Casp11* (**k, s**), *Il6* (**l, t**), *Ifna* (**m, u**), *Ifnb* (**n, v**), *Tnfa* ***(o, x)***. Data are presented as mean values ± SD.

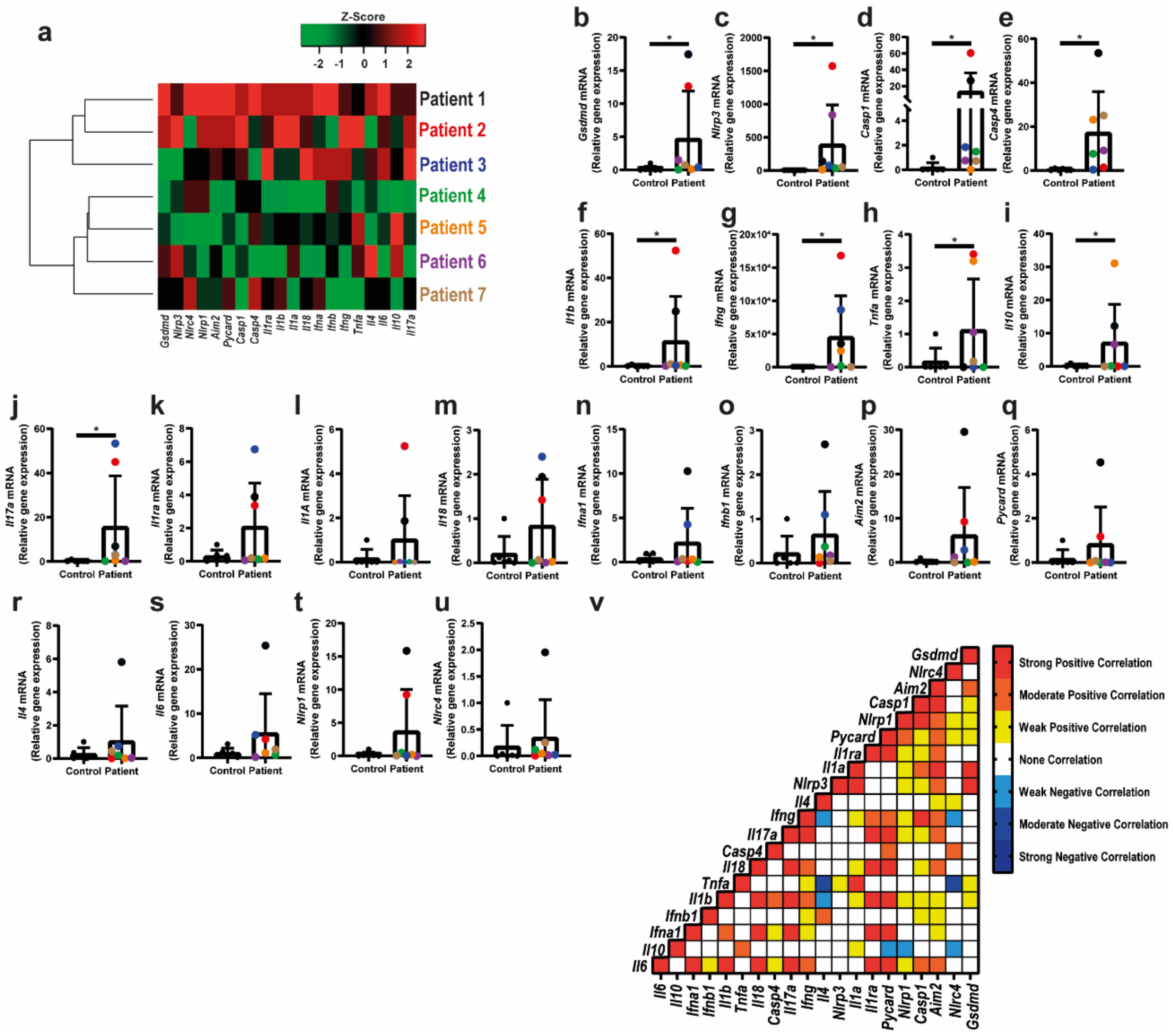

Supplementary Fig. 6. Inflammasome and inflammatory cytokines expression in skin biopsies of patients with cutaneous leishmaniasis. (**a**) Heatmap of the mRNA expression of inflammasome, inflammatory molecules, and cytokine in skin biopsies of 7 patients with cutaneous leishmaniasis. (**b-u**) Expression of mRNA in the skin biopsies of cutaneous leishmaniasis and healthy controls (skin of individuals who underwent reductive mastectomy). Selected genes were *Gsdmd* (**b**), *Nlrp3* (**c**), *Casp1* (**d**), *Casp4* (**e**), *Il1b* (**f**), *Ifng* (**g**), *Tnfa* (**h**), *Il10* (**i**), *Il17a* (**j**), *Il1ra* (**k**), *Il1a* (**l**), *Il18* (**m**), *Ifna1* (**n**), *Ifnb1* (**o**), *Aim2* (**p**), *Asc* (**q**), *Il4* (**r**), *Il6* (**s**), *Nlrp1* (**t**), *Nlrc4* (**u**). Data are presented as mean values ± SD. Correlation matrix of inflammasome gene expression in skin lesion biopsies of patients with cutaneous leishmaniasis (**v**).

Supplementary Table 1. Leishmaniasis patients' characteristics

**Demographics Characteristics**	**Leishmaniasis patients** Mean (±SD) or n(%)
N	7
Age (years)	52.75 (±12.45)
Sex	
Male	6 (85.71%)
Female	1 (14.28%)
L. braziliensis strain	7 (100.00%)
**Treatment**	
Glucantine	4 (57.14%)
Pentamidina	1 (14.28%)
Liposomal Amphotericin B + Glucantine	1 (14.28%)
Miltefosine + Conventional Amphotericin B + Glucantine	1 (14.28%)
**Laboratorial findings**	
Montenegro skin test	
Positive	2 (28.57%)
Negative	2 (28.57%)
Unperformed	3 (42.85%)
HIV positive	1 (14.28%)
HIV negative	6 (85.71%)
Hemoglobin (g/dL)	13.05 (±2.56)
White blood cells (10^3/ul)	5.76 (±2.57)
Neutrophil (%)	49.35 (±7.97)
Lymphocytes (%)	37.73 (±6.96)
Monocyte (%)	7.58 (±1.84)
ALT (IU/L)	32.51 (±17.42)
AST (IU/L)	34.16 (±9.71)
Alkaline phosphatase (U/L)	232.09 (±126.18)
Cholesterol (mg/dL)	170.23 (±43.96)
Blood glucose (mg/dL)	122.46 (±34.64)
Creatinine (mg/dL)	1.04 (±0.32)
Urea (mg/dL)	26.88 (±17.43)
**Histopathological findings**	
Lesion size (cm)	6 (±2.94)
Inflammatory infiltrate	7 (100.00%)
Acanthosis	5 (71.42%)
Compact hyperkeratosis	2 (28.57%)
Spongiosis	2 (28.57%)
Ulcerated epidermis	4 (57.14%)
Amastigotes	5 (71.42%)

Supplementary Table 2. The list of primer sequences for real-time PCR

Gene	Sequence of primer (5'- 3')
Human *Gsdmd*	F: ATGAGGTGCCTCCACAACTTCC
	R: CCAGTTCCTTGGAGATGGTCTC
Human *Nlrp3*	F: GGACTGAAGCACCTGTTGTGCA
	R: TCCTGAGTCTCCCAAGGCATTC
Human *Il1a*	F: TGTATGTGACTGCCCAAGATGAAG
	R: AGAGGAGGTTGGTCTCACTACC
Human *Il1ra*	F: ATGGAGGGAAGATGTGCCTGTC
	R: GTCCTGCTTTCTGTTCTCGCTC
Human *Nlrc4*	F: AGGTCCCACAACTCGTCAAGCT
	R: TGCTCACACGATTTCCCGCCAA
Human *Pycard*	F: AGCTCACCGCTAACGTGCTGC
	R: GCTTGGCTGCCGACTGAGGAG
Human *Nlrp1*	F: ATTGAGGGCAGGCAGCACAGAT
	R: CTCCTTCAGGTTTCTGGTGACC
Human *Casp1*	F: GCTGAGGTTGACATCACAGGCA
	R: TGCTGTCAGAGGTCTTGTGCTC
Human *Aim2*	F: GCTGCACCAAAAGTCTCTCCTC
	R: CTGCTTGCCTTCTTGGGTCTCA
Human *Il6*	F: AGACAGCCACTCACCTCTTCAG
	R: TTCTGCCAGTGCCTCTTTGCTG
Human *Gapdh*	F: GTCTCCTCTGACTTCAACAGCG
	R: ACCACCCTGTTGCTGTAGCCAA
Human *Tnf Alpha*	F: CTCTTCTGCCTGCTGCACTTTG
	R: ATGGGCTACAGGCTTGTCACTC
Human *Il10*	F: TCTCCGAGATGCCTTCAGCAGA
	R: TCAGACAAGGCTTGGCAACCCA
Human *Il1b*	F: CCACAGACCTTCCAGGAGAATG
	R: GTGCAGTTCAGTGATCGTACAGG
Human *Il18*	F: GATAGCCAGCCTAGAGGTATGG
	R: CCTTGATGTTATCAGGAGGATTCA
Human *Casp4*	F: GGGATGAAGGAGCTACTTGAGG
	R: CCAAGAATGTGCTGTCAGAGGAC
Human *Il17a*	F: CGGACTGTGATGGTCAACCTGA
	R: GCACTTTGCCTCCCAGATCACA
Human *Ifng*	F: GAGTGTGGAGACCATCAAGGAAG
	R: TGCTTTGCGTTGGACATTCAAGTC
Human *Ifnb1*	F: CTTGGATTCCTACAAAGAAGCAGC
	R: TCCTCCTTCTGGAACTGCTGCA
Human *Ifna1*	F: AGAAGGCTCCAGCCATCTCTGT
	R: TGCTGGTAGAGTTCGGTGCAGA
Human *Il4*	F: CCGTAACAGACATCTTTGCTGCC
	R: GAGTGTCCTTCTCATGGTGGCT
Mouse *Il1b*	F: TGGACCTTCCAGGATGAGGACA
	R: GTTCATCTCGGAGCCTGTAGTG

Mouse *Il18*	F: GACAGCCTGTGTTCGAGGATATG
	R: TGTTCTTACAGGAGAGGGTAGAC
Mouse *Casp1*	F: GGCACATTTCCAGGACTGACTG
	R: GCAAGACGTGTACGAGTGGTTG
Mouse *Casp11*	F: GTGGTGAAAGAGGAGCTTACAGC
	R: GCACCAGGAATGTGCTGTCTGA
Mouse *IL6*	F: TACCACTTCACAAGTCGGAGGC
	R: CTGCAAGTGCATCATCGTTGTTC
Mouse *Ifna1*	F: GGATGTGACCTTCCTCAGACTC
	R: ACCTTCTCCTGCGGGAATCCAA
Mouse *Ifnb1*	F: GCCTTTGCCATCCAAGAGATGC
	R: ACACTGTCTGCTGGTGGAGTTC
Mouse *Tnfa*	F: GGTGCCTATGTCTCAGCCTCTT
	R: GCCATAGAACTGATGAGAGGGAG
Mouse *Gapdh*	F: CATCACTGCCACCCAGAAGACTG
	R: ATGCCAGTGAGCTTCCCGTTCAG
