# Supplementary material for: Gasdermin-D activation promotes NLRP3 activation and host resistance to Leishmania infection
Source: Nat Commun. 2023 Feb 24;14:1049. doi: 10.1038/s41467-023-36626-6 (PMC9958042; doi:10.1038/s41467-023-36626-6)
Supplement: Supplementary file 2 — Description of additional supplementary files [file 41467_2023_36626_MOESM2_ESM.pdf]

Gasdermin-D activation promotes NLRP3 activation and host resistance to *Leishmania* infection

Keyla S.G. de Sá et al.

Description of additional supplementary files:

Supplementary Movie 1.

Non-infected macrophages. Movie of BMDMs from C57BL/6 mice pretreated for 4h with LPS (100 ng/mL) and left uninfected. Propidium iodide (5µg/ml) was added to the culture medium. Images were captured every 10 min by BioStation IM-Q microscopy with a 40x objective for 20 hours.

Supplementary Movie 2.

Nigericin-treated macrophages. Movie of BMDMs from C57BL/6 mice pretreated for 4h with LPS (100 ng/mL) and treated with nigericin (10µM). Propidium iodide (5µg/ml) was added to the culture medium. Images were captured every 10 min by BioStation IM-Q microscopy with a 40x objective for 20 hours.

Supplementary Movie 3.

***Leishmania*-infected macrophages.** Movie of BMDMs from C57BL/6 mice pretreated for 4h with LPS (100 ng/mL) and infected with *L. amazonensis* with MOI 10. Propidium iodide (5µg/ml) was added to the culture medium. Images were captured every 10 min by BioStation IM-Q microscopy with a 40x objective for 20 hours.
